# European Headache Federation (EHF) critical re-appraisal and meta-analysis of oral drugs in migraine prevention—part 1: amitriptyline

**DOI:** 10.1186/s10194-023-01573-6

**Published:** 2023-04-11

**Authors:** Christian Lampl, Jan Versijpt, Faisal Mohammad Amin, Christina I. Deligianni, Raquel Gil-Gouveia, Tanvir Jassal, Antoinette MaassenVanDenBrink, Raffaele Ornello, Jakob Paungarttner, Margarita Sanchez-del-Rio, Uwe Reuter, Derya Uluduz, Tessa de Vries, Dena Zeraatkar, Simona Sacco

**Affiliations:** 1 Department of Neurology and Stroke Unit, Konventhospital Barmherzige Brüder Linz, Linz, Austria; 2 Headache Medical Center Linz, Linz, Austria; 3 Department of Neurology, Vrije Universiteit Brussel (VUB), Universitair Ziekenhuis Brussel (UZ Brussel), Brussels, Belgium; 4 Danish Headache Center, Department of Neurology, Rigshospitalet Glostrup, University of Copenhagen, Copenhagen, Denmark; 5 Department of Neurology, Athens Naval Hospital, Athens, Greece; 6 Hospital da Luz Headache Center, Neurology Department, Hospital da Luz Lisboa, Lisbon, Portugal; 7 Center for Interdisciplinary Research in Health, Universidade Católica Portuguesa, Lisbon, Portugal; 8 Department of Anesthesia and Department of Health Research Methods, Evidence and Impact, McMaster University, Hamilton, Canada; 9 Department of Internal Medicine, Erasmus MC Medical Center, Rotterdam, The Netherlands; 10 Department of Biotechnological and Applied Clinical Sciences, University of L’Aquila, L’Aquila, Italy; 11 Department of Neurology, Clinica Universidad de Navarra, Madrid, Spain; 12 Department of Neurology, Charité Universitätsmedizin Berlin, Berlin, Germany; 13 Department of Neurology Istanbul Cerrahpasa Medical Faculty, Istanbul, Turkey

**Keywords:** Migraine, Amitriptyline, Prophylactic treatment, Meta-analysis

## Abstract

**Objective:**

The aim of this paper is to critically re-appraise the published trials assessing amitriptyline for migraine prophylaxis.

**Methods:**

We report our methods and results following the Preferred Reporting Items for Systematic Reviews (PRISMA), by searching MEDLINE, EMBASE, Cochrane CENTRAL, and ClinicalTrials.gov for randomized trials of pharmacologic treatments for migraine prophylaxis. We included randomized trials that compared amitriptyline with placebo for migraine prophylaxis in adults. Our outcomes of interest were informed by the Outcome Set for preventive intervention trials in chronic and episodic migraine (COSMIG) and include the proportion of patients who experience a 50% or more reduction in migraine days per month, migraine days per month, and adverse events leading to discontinuation.

We assessed risk of bias by using a modified Cochrane RoB 2.0 tool and the certainty of evidence by using the GRADE approach.

**Results:**

Our search yielded 10.826 unique records, of which three trials (*n* = 622) were eligible for data synthesis and analysis. We found moderate certainty evidence that amitriptyline increases the proportion of patients who experience a 50% or more reduction in monthly migraine days, compared to placebo (relative risk: 1.60 (95% CI 1.17 to 2.19); absolute risk difference: 165 more per 1,000 (95% CI 47 more to 327 more). We found moderate certainty evidence that amitriptyline increases the proportion of patients who discontinue due to adverse events compared to placebo (risk difference: 0.05 (95% CI 0.01 to 0.10); absolute risk difference: 50 more per 1,000 (95% CI 10 more to 100 more).

**Conclusions:**

Our meta-analysis showed that amitriptyline may have a prophylactic role in migraine patients, however these results are far from robust. This warrants further large-scale research to evaluate the role of amitriptyline in migraine prevention.

**Supplementary Information:**

The online version contains supplementary material available at 10.1186/s10194-023-01573-6.

## Introduction

**Fig. 1:**
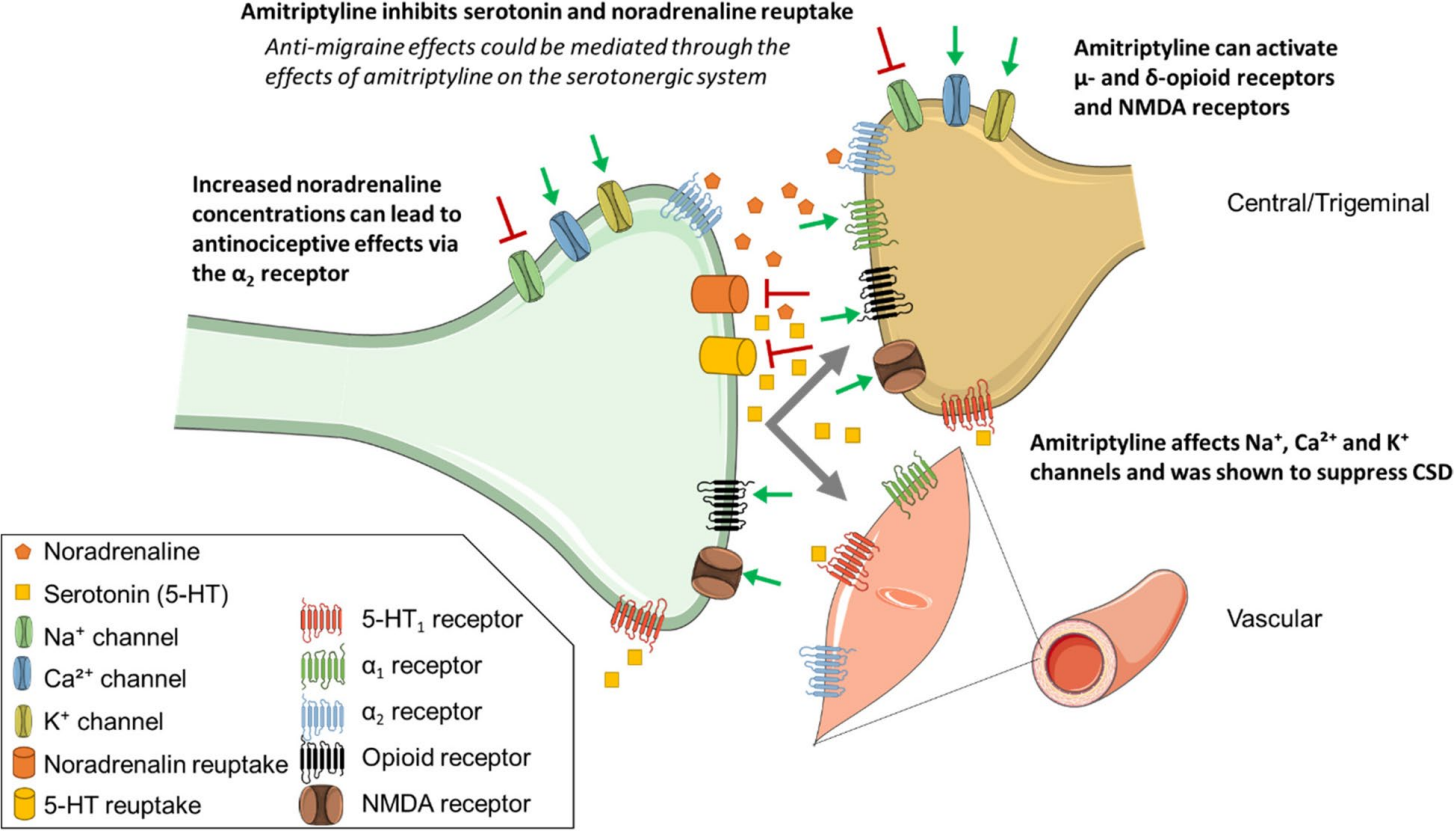
Potential mechanisms of action for the anti-migraine effect of the tricyclic antidepressant amitriptyline. Amitriptyline inhibits the uptake of serotonin and noradrenaline in the synaptic cleft, and possibly exerts its antimigraine effects by affecting serotonergic transmission or through antinociceptive effects via activation of the α2 adrenoreceptor [8]. In addition, tricyclic antidepressants affect sodium [14] calcium [10] and potassium [11] channels, exert an effect on adrenergic α1, NMDA and opioid receptors [12] and suppress cortical spreading depression (CSD), which could be underlying migraine aura [13]

Migraine is a highly disabling disease that often requires preventive treatment, especially in highly frequent episodic and chronic migraine. Patients need prophylactic drugs to reduce the migraine burden, either to decrease the occurrence of acute attacks and/or the need of analgesics. All older prophylactic drugs that are used in migraine have been developed for other indications and were later found effective in migraine. Tricyclic antidepressants (TCAs) were among the first medications identified as having a preventive benefit for migraine. Amitriptyline was discovered in the late 1950s and was approved by the U.S. Food and Drug Administration (FDA) in 1961. The beneficial use of amitriptyline in migraine was first reported in the late 1960s by Friedman [1] and Mahloudji [2]. Studies of migraine preventive use in the USA show that TCAs are the second most prescribed medications for migraine prevention, after topiramate [3]. Amitriptyline is considered as a level B drug for migraine prophylaxis by the American Headache Society (AHS) and American Academy of Neurology (AAN), meaning it is regarded as "probably effective” even though it has not been approved by the FDA for the prophylactic use in migraine [4]. In Europe, amitriptyline is considered as a ‘drug of second choice’ [5]. The exact mechanism of action of amitriptyline in migraine prophylaxis is unclear. The neurotransmitter 5-hydroxytryptamine (5-HT, serotonin) is involved in migraine pathophysiology [6] and the acute antimigraine medication class of triptans targets the 5-HT receptor subtypes 5-HT_1B/1D/(1F)_ [7]. TCAs inhibit the uptake of 5-HT in the synaptic cleft [8] so it is likely that the antimigraine effect of amitriptyline results from its effects on serotonergic transmission. Moreover, inhibition of reuptake of noradrenaline leads to increased concentrations of this neurotransmitter in the synaptic cleft, which could exert antinociceptive effects through activation of α_2_-adrenoreceptors [8, 9]. In addition to 5-HT and noradrenaline reuptake inhibition, TCAs have multiple other targets, including anticholinergic and antihistaminergic effects, they affect sodium, calcium [10] and potassium channels [11], and exert an effect on adrenergic α_1_-adrenoreceptors, N-methyl-D-aspartate (NMDA) and opioid receptors [12]. In a rat model, amitriptyline was shown to suppress cortical spreading depression (CSD), which is thought to be the underlying mechanism of migraine aura [13]. These many sites of action could potentially contribute to the antimigraine effect of amitriptyline (Fig. 1) [14], but they also relate to the various adverse effects caused by this drug.

The aim of this paper is to critically re-appraise the published trials assessing amitriptyline for migraine prophylaxis. We focus on amitriptyline because, compared to other antidepressants, it is the most widely studied for migraine and thus has the largest evidence base supporting its efficacy and safety for migraine.

## Methods

We report our methods and results following the Preferred Reporting Items for Systematic Reviews (PRISMA) [15].

### Search strategy (Supplement 1)

In consultation with an experienced research librarian, we searched MEDLINE, EMBASE, Cochrane CENTRAL, and ClinicalTrials.gov from inception to August 13, 2022 for randomized trials of pharmacologic treatments for migraine prophylaxis, without language restrictions. We supplemented our search by retrieving references of similar systematic reviews and meta-analyses [16].

### Screening and study eligibility

Following training and calibration exercises to ensure sufficient agreement, pairs of reviewers, working independently and in duplicate, reviewed titles and abstracts of search records and subsequently the full texts of records deemed potentially eligible at the title and abstract screening stage. Reviewers resolved discrepancies by discussion, or, when necessary, by adjudication with a third viewer. We included randomized trials that compared amitriptyline with placebo for migraine prophylaxis in adults. We excluded trials that investigated abortive rather than prophylactic interventions and trials that randomized children or adolescents. We excluded trials that randomized fewer than 25 participants as we anticipated that smaller trials may be unrepresentative and at higher risk of publication bias [17].

### Data extraction

Following training and calibration to ensure sufficient agreement, pairs of reviewers, working independently and in duplicate, extracted data from eligible studies. Reviewers resolved discrepancies by discussion and if necessary, by adjudication with a third party. We extracted trial characteristics, patient characteristics, diagnostic criteria, type of migraine, intervention characteristics, and outcomes of interest at the longest reported follow-up time at which patients were still using the interventions being investigated. Our outcomes of interest were informed by the Outcome Set for preventive intervention trials in chronic and episodic migraine (COSMIG) and include the proportion of patients who experience a 50% or more reduction in migraine days per month, migraine days per month, and adverse events leading to discontinuation [18]. We prioritized extracting monthly migraine days when reported but also extracted monthly headache days or monthly migraine attacks when monthly migraine days were not reported.

### Risk of bias assessments

Following training and calibration to ensure sufficient agreement, reviewers working independently and in duplicate, assessed risk of bias using a modified Cochrane RoB 2.0 tool [19, 20]. For each trial, we rated each outcome as either ‘low risk of bias’, ‘some concerns –probably low risk of bias’, ‘some concerns –probably high risk of bias’, and ‘high risk of bias’ across the following domains: bias arising from the randomization process, bias due to departures from the intended intervention, bias due to missing outcome data, bias in measurement of the outcome, and bias in selection of the reported results. Reviewers resolved discrepancies by discussion and if necessary, by adjudication with a third party.

### Data synthesis and analysis

For all outcomes, we performed frequentist random-effects meta-analysis using the restricted maximum likelihood (REML) estimator [21]. We also performed sensitivity analyses using the Paule-Mandel heterogeneity estimator. We analyzed 50% or more reduction in monthly migraine days as relative risks, monthly migraine days as mean differences, and adverse events leading to discontinuation as risk differences, since we expected many studies to report no or few events with placebo. To facilitate interpretation, we report dichotomous outcomes as number of events per 1,000 patients. We summarize heterogeneity using the I^2^ statistic and interpret an I^2^ value of 0% to 40% as not important, 30% to 60% as moderate heterogeneity, 50% to 90% as substantial heterogeneity, and 75% to 100% considerable heterogeneity [22].

We anticipated that the effects of treatments may vary based on risk of bias, baseline monthly migraine days, and the proportion of patients that had previously used prophylactic therapy. To test for subgroup effects based on these factors, we performed pairwise meta-regressions comparing results rated at low versus high risk of bias and trials below versus above the median number of monthly migraine days or proportion of patients that had previously used prophylactic therapy. We assessed the credibility of subgroup effects using the ICEMAN tool [23]. For analyses with 10 or more studies, we planned to test for publication bias by visually inspecting funnel plots and Eggers tests [24]. We performed all analyses using the *meta* and *metafor* packages in R (version 4.1.2) [25, 26].

### Assessment of the certainty (quality) of evidence

We assessed the certainty of evidence using the GRADE approach [27]. For each outcome, we rated certainty of each comparison as either high, moderate, low, or very low based on risk of bias, inconsistency, indirectness, imprecision, and publication bias. We made judgements of imprecision using the minimally contextualized approach [28]. The minimally contextualised approach considers only whether confidence intervals include the null effect and thus does not consider whether plausible effects, captured by confidence intervals, include both important and trivial effects. To evaluate the certainty of no effect, we used minimally important differences, sourced from the literature and by consensus from the authors. Because we were unable to source established minimally important differences for migraine from the literature, based on consensus of the authors, we considered a 15% increase in the proportion of patients who experienced a 50% or more reduction in monthly migraine days, a reduction of 2 monthly migraine days, and a 2% increase in patients who experienced adverse events leading to discontinuation as minimally important. We report results using GRADE simple language summaries (i.e., describing high certainty evidence with declarative statements, moderate certainty evidence with ‘probably’, low certainty evidence with ‘may’ and very low indicated by ‘very uncertain’) [29].

## Results

**Fig. 2:**
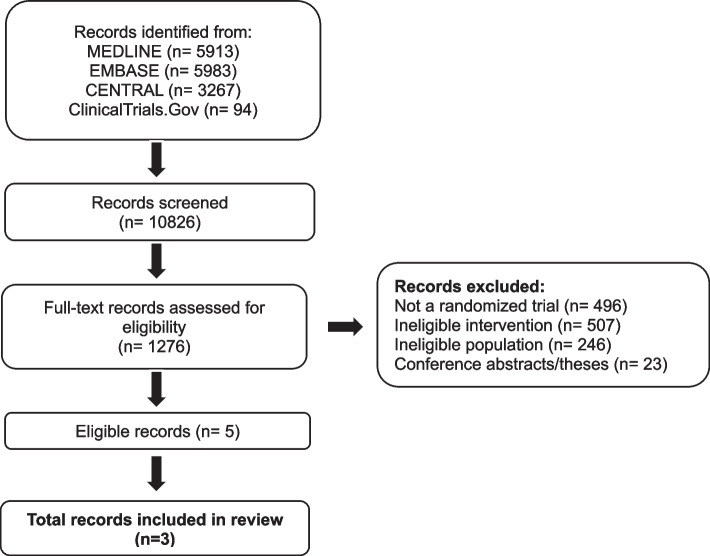
Selection of studies for the systematic review. Our search yielded a total of 10,826 unique records. After title and abstract screening 1,276 records proved potentially eligible and after full-text review 5 records proved eligible. We excluded records if they did not describe full-text peer-reviewed reports of randomized trials that compared amitriptyline with placebo for prophylaxis of migraine in adult patients

Our search yielded 10.826 unique records, of which five trials were eligible for the narrative description [30–34] and three for data synthesis and analysis [31–33]. Figure 2 presents details about study selection.

### Narrative description of amitriptyline in placebo-controlled trials

In the first clinical trial, published by Gomersall and Stuart in 1973 [30], amitriptyline (10–60 mg per day) reduced the number of migraine attacks by 42% (*p* < 0.001), in about half of the subjects by > 50%. However, only 20 subjects of 26 who initiated did complete the trial.

The Couch and Hassanein (1979) trial used a composite migraine score including frequency, severity, and duration of attacks as the primary outcome parameter for efficacy [31]. This specific score was reduced by more than 50% in 55% of the amitriptyline-treated patients (dose up to 100 mg per day), compared with 34% of the placebo-treated patients. The therapeutic gain in that study was 21%. Data on migraine frequency were not presented, and patients with comorbid depression were not excluded.

In another placebo-controlled trial published in 2005 the prophylactic activity of propranolol and amitriptyline on frequency, duration and severity of migraine attacks was compared in 105 patents. Amitriptyline (25 mg twice per day) significantly reduced the frequency, duration and intensity of migraine attacks after treatment of 45 days [32]. After discontinuation, the rebound effect was higher than in the propranolol group.

Couch published an analysis of a trial that was performed between 1976 and 1979 subsequently in 2011 [33]. 391 subjects with migraine and chronic daily headache were included. There was a significant improvement in headache frequency for amitriptyline 25 mg over placebo at 8 weeks (*p* 0.018) but not at 12, 16, or 20 weeks. There were no significant differences in headache severity or duration between amitriptyline and placebo at any time point during the study. The drop-out rate was 52% at week 20.

Another placebo-controlled trial with 196 patients randomized to receive either melatonin as active comparator or amitriptyline was published in 2016 [34]. Amitriptyline 25 mg was superior to placebo (*p* < 0.05) for reducing migraine days per month after 12 weeks compared to baseline but not superior to melatonin. Melatonin was better than amitriptyline for the secondary endpoint (50% responder rate) and was better tolerated than amitriptyline.

### Data synthesis and analysis

**Fig. 3:**
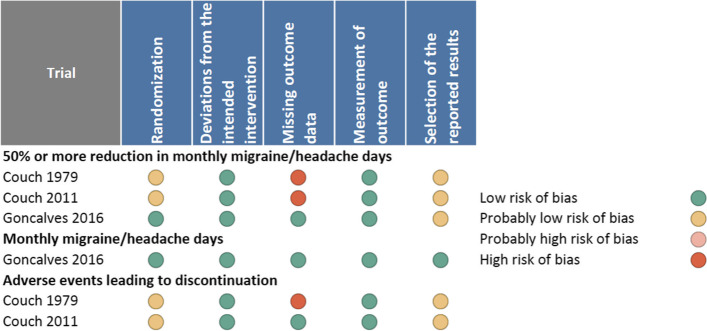
Risk of bias ratings. Two out of three trials and one out of two trials were at high risk of bias due to missing outcome data for 50% or more reduction in monthly migraine days and adverse events leading to discontinuation, respectively. One trial, reporting on monthly migraine days, was at low risk of bias

We included three trials in our quantitative analysis, including 622 patients [31, 33, 34]. We excluded one trial from the quantitative analysis since it included only 20 participants [30] and one other trial [32] because it only reported total number of participants and not the number of participants in each arm, with migraine attacks and not migraine/headache days as primary outcome parameter which precludes analysis. Two of the three trials were industry-funded and performed in the USA [31, 33] and the third trial was funded by a public grant from Brazil [34]. More than three quarters of patients were middle-aged women. Two trials recruited patients with a minimum of two migraine days per month [31, 33] and one trial recruited patients with a minimum of 4 migraine days per month and a maximum of 15 headache days per month [34]. Table 1 presents the trial characteristics and Fig. 3 presents the risk of bias ratings.

**Table 1:** Trial characteristics

Study	Registration	Funding	Country	Mean age	% Male	Duration of migraine (years)	% Aura	Migraine days per month at baseline	Interventions	Number of patients
Couch 1979 [30]	NR	Merck Laboratories	US	NR	16.0	NR	NR	NR	Amitriptyline 100 mg/day or MTD, titrated over 4 weeks, maintained for 4 weeks, oral Placebo	100
Couch 2011 [32]	NR	Merck, Sharp, and Dohme Research Laboratories	US	34.9	19.0	NR	NR	NR	Amitriptyline 100 mg/day or MTD, titrated over 4 weeks, maintained for 12 weeks, oral Placebo	391
Gonçalves 2016 [33]	NCT 01357031	Fundação de Amparo a Pesquisa de São Paulo	Brazil	36.9	24.6	22.2	16.1	7.3	Amitriptyline 25 mg/day for 12 weeks Placebo	131

*NR* not reported, *NCT* number clinical trial, *MTD* maximum tolerable dose

### 50% responder rate

**Fig. 4:**
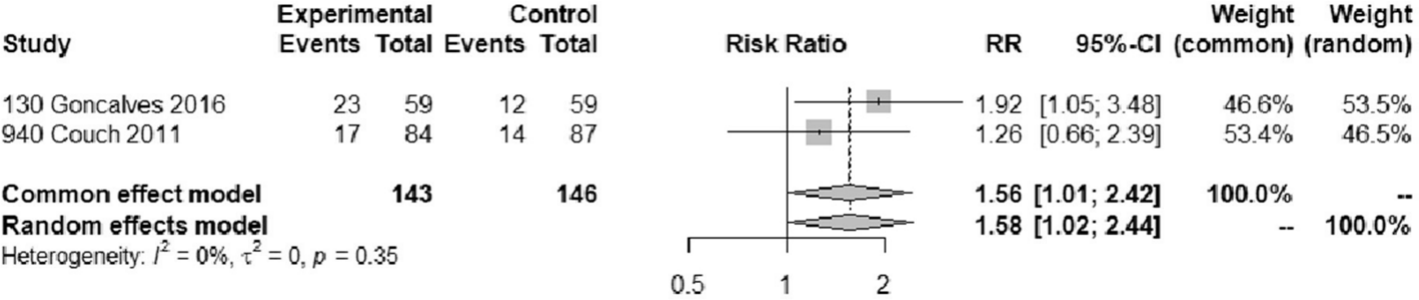
Sensitivity analysis of analysis for 50% or more reduction in monthly migraine days excluding a trial that reported on a 50% reduction in a migraine score

**Fig. 5:**
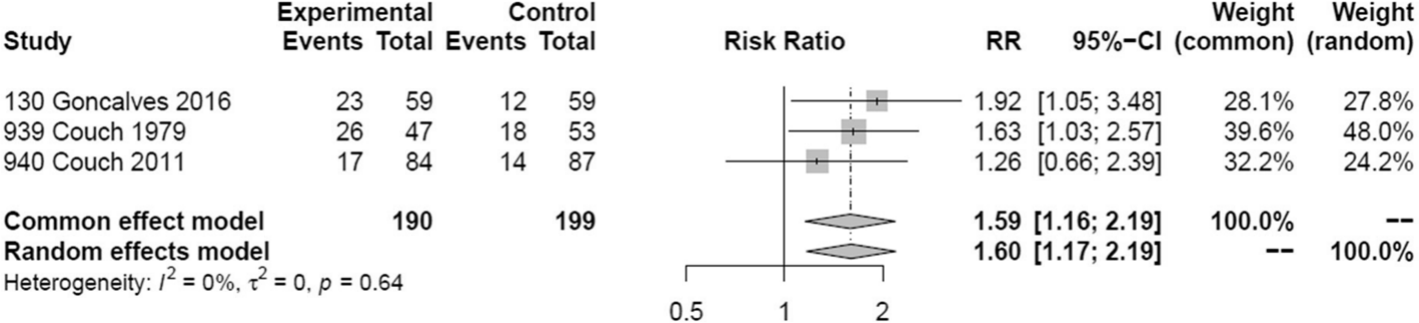
The forest plot shows pooled relative risk and associated confidence intervals comparing 50% or more reduction in monthly migraine days for amitriptyline versus placebo

**Fig. 6:**
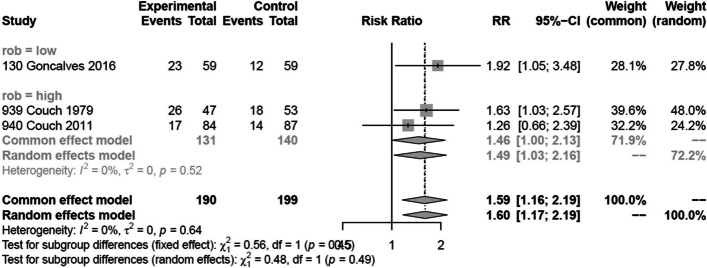
Subgroup analysis comparing results of trials at low vs. high risk of bias for 50% responder rate

Two trials [33, 34] reported on 50% or more reduction in monthly migraine days in 289 patients and one trial [31] reported on 50% responder rate in 100 patients. We performed a sensitivity analysis excluding the trial that reported responder rate. The sensitivity analysis produced results consistent with the main analysis (Fig. 4). Two out of three trials were rated at high risk of bias, due to missing outcome data (Fig. 3). Two of the trials also failed to describe methods for allocation concealment. We were unable to make confident judgements about potential for selective reporting due to lack of publicly accessible protocol or registration files for two trials — likely since these trials were performed/published before trial registration practices became common. We found moderate certainty evidence that amitriptyline probably increases the proportion of patients who experience a 50% or more reduction in monthly migraine days, compared to placebo (Table 2; Figs. 4 and 5). The certainty of evidence was downgraded by one level due to concerns about risk of bias. We anticipated that the effects of amitriptyline may be different based on risk of bias (i.e., low vs. high risk of bias), mean monthly migraine days at baseline, and the proportion of patients who reported having previously used prophylactic drugs and had planned to perform subgroup analyses investigating the effects of these variables on results. Due to lack of reporting of mean monthly migraine days at baseline and the proportion of patients who had previously used prophylactic drugs, we were unable to perform subgroup analyses addressing these factors. The subgroup analysis based on risk of bias did not suggest that the trial at low risk of bias produced results that were different from the trial at high risk of bias (Fig. 6). A sensitivity analysis using the Paule-Mandel heterogeneity estimator yielded results consistent with the primary analysis (Supplement 2).

**Table 2:** Amitriptyline compared to placebo for migraine prophylaxis

Patient or population: migraine Intervention: prophylaxis with amitriptyline Comparison: placebo					
Outcomes	№ of participants (studies)	Certainty of the evidence (GRADE)	Relative effect (95% CI)	Anticipated absolute effects^a^	
				Risk with placebo	Risk difference with Amitriptyline
50% or more reduction in monthly migraine days	389 (3 RCTs)	Moderate (downgraded due to risk of bias)	RR 1.60 (1.17 to 2.19)	275 per 1,000	165 more per 1,000 (47 more to 327 more)
Monthly migraine days	118 (1 RCT)	High	-	NA	MD 1.2 migraine days fewer (2.1 fewer to 0.3 fewer)
Adverse events leading to discontinuation	507 (2 RCTs)	Moderate (downgraded due to risk of bias)	RD 0.05 (0.01 to 0.10)	0 per 1,000	50 more per 1,000 (10 more to 100 more)

*CI* confidence interval, *MD* mean difference, *RR* risk ratio, *RD* Risk difference

GRADE Working Group grades of evidence

High certainty: we are very confident that the true effect lies close to that of the estimate of the effect

Moderate certainty: we are moderately confident in the effect estimate: the true effect is likely to be close to the estimate of the effect, but there is a possibility that it is substantially different

Low certainty: our confidence in the effect estimate is limited: the true effect may be substantially different from the estimate of the effect

Very low certainty: we have very little confidence in the effect estimate: the true effect is likely to be substantially different from the estimate of effect

^a^The risk in the intervention group (and its 95% confidence interval) is based on the assumed risk in the comparison group and the relative effect of the intervention (and its 95% CI)

### Monthly migraine days

Only one trial, including 118 patients, reported on the reduction in monthly migraine days [34]. The trial was rated at low risk of bias (Fig. 3). We found high certainty evidence that amitriptyline reduces monthly migraine days (Table 2). We were unable to perform subgroup analyses based on risk of bias, mean monthly migraine days at baseline, and the proportion of patients who reported having previously used prophylactic drugs due to too few trials.

### Adverse events leading to discontinuation

**Fig. 7:**
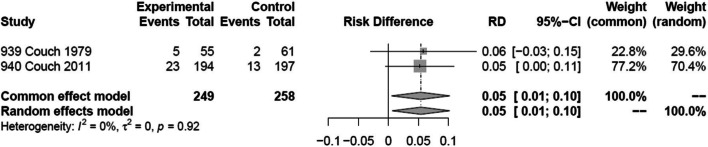
Forest plot showing meta-analysis comparing amitriptyline with placebo for adverse events leading to discontinuation

Two trials, including 507 patients, reported on adverse events leading to discontinuation [31, 33]. One of the two trials was rated at high risk of bias due to missing outcome data [30]. We found moderate certainty evidence that amitriptyline probably increases the proportion of patients who discontinue due to adverse events compared to placebo. The certainty of evidence was downgraded by one level due to risk of bias (Fig. 7). We were unable to perform subgroup analyses based on risk of bias, mean monthly migraine days at baseline, and the proportion of patients who reported having previously used prophylactic drugs due to too few trials. A sensitivity analysis using the Paule-Mandel heterogeneity estimator yielded results consistent with the primary analysis (Supplement 1).

Only one trial reported specific adverse events that led to discontinuation, which included rash, hypertension, nausea, and numbness of hands and feet [30].

## Discussion

Amitriptyline is widely used in the prophylactic treatment of migraine. Our meta-analysis showed that the tricyclic antidepressant amitriptyline may have a prophylactic role in migraine patients, however, in view of the studies retrieved and included in our meta-analysis, these results are far from robust. This warrants further large-scale research to evaluate the role of amitriptyline in migraine prevention. As it is in guidelines, it is often used in the real-life setting. An adequate registry would be able to collect relevant information on its role in migraine management. The most important adverse effects of amitriptyline are drowsiness and anticholinergic symptoms such as dry mouth, constipation, and tachycardia. Weight gain occurs in many patients together with elevated levels of leptin, insulin, and C peptide [35], and can be a limiting factor leading to impaired compliance and discontinuation. Occasionally, amitriptyline may provoke glaucoma, PQ and QT interval prolongation on electrocardiogram (ECG), as well as benign prostate hypertrophy. Amitriptyline is metabolized by cytochrome P450 (CYP) isoenzymes, particularly CYP2D6, which is responsible for multiple interactions [36]. So far, three placebo-controlled trials found amitriptyline significantly better than placebo at reducing a headache index or frequency, but the magnitude of effect, albeit significant as compared to placebo is limited. Furthermore, the trial by Couch published in 2011 with patient enrollment initiation between 1977 and 1979 showed that amitriptyline was superior to placebo in migraine prophylaxis at 8 weeks but, because of a robust placebo response, not at subsequent time points. Therefore, this study must be rated as negative.

There are many limitations in the described trials, that have to be raised and critically analyzed. Some of them are listed below: (i) baseline observation period: was this prospective or historically driven? Was baseline attack frequency measured by a standardized questionnaire or not? If not, then this is extremely susceptible to bias. (ii) blinding: how was blinding performed and maintained, especially during the titration phase? Can there be unblinding, e.g. due to side effects that can be quite pronounced at the high doses of amitriptyline used? (iii) analysis: was the analysis of the primary endpoint prospectively determined or was there the possibility of a retrospective interpretation and selection of only the so-called positive endpoints? (iv) outcomes: what was the primary endpoint? Was it and the time of assessment predetermined or was the most positive endpoint only selected at variable time points after the trial? (v) dropout rate: how were the results adjusted for dropouts? How were dropouts handled? (vi) one of the trials was conducted in the 1970s, but not published until 2011 [33]. This is highly unusual, and raises questions on the solidity of the data, unless one could study the original raw data. (vii) how where different types of headaches diagnosed and discriminated? Amitriptyline is effective in tension-type headache, and many patients have a combination of both tension-type headache and migraine [37], which complicates effect assessment and interpretation if the inclusion and end-point definition are too vague and include both headache types.

Taken together, the quality of the studies included in the current meta-analysis is questionable. Nevertheless, one guideline recommends amitriptyline as first line agent with a dose range between 30 and 150 mg with a medium to high efficacy and mild or infrequent side effects [38]. According to the 2012 published guidelines for preventing episodic migraine (defined as headaches that occur fewer than 15 times per month) established by the American Headache Society (AHS) and the American Academy of Neurology (AAN), amitriptyline is a level B medication for migraine prophylaxis, meaning it is regarded as "probably effective [39]. In the 2009 revised European guidelines on the drug treatment of migraine, amitriptyline is recommended as drug of 2^nd^ choice for migraine prophylaxis [5]. Besides these recommendations there is still a need for further clinical trials in individuals of all ages, since it is still based on old trials with small numbers of participants, different treatment endpoints and old regulatory approval standards.

Nowadays, based on standards from Food and Drug Administration (FDA) and European Medicines Agency (EMA), drugs do not get approved without at least two well designed positive placebo-controlled trials (https://www.fda.gov/drugs/development-approval-process-drugs). Some of the trials considered in this review had limited sample size, which leaves the findings unclear for several outcome measures. Length of follow-up was often too short (mean length, 12 weeks; recommended, 24 weeks), and the clinical outcomes measured (scales or indices) often did not have a well-established rationale and were not prespecified. The appropriateness of statistical analyses was a frequent matter of concern, particularly considering multiple treatment comparisons, repeated measurements over time, and questionable subgroup analyses.

Another heterogeneity is the fact that some of the presented studies examined migraine preventive efficacy only in those patients without concomitant depression, whereas others allowed concurrent depression. In the past few years, the association between migraine and depression has been described in both clinic- and community-based populations [40]. Many researchers maintain that chronic migraine pain can induce a reactive depression that becomes more evident the more chronic the pain is. To explain a development from migraine to depression, it has been hypothesized that unpredictable attacks of severe pain might lead to anxiety and depression. However, in longitudinal studies, the evidence supports a bidirectional relationship between migraine and depression, with each disorder increasing the risk of the other [41, 42]. In such cases, amitriptyline may provide more benefit than other drugs. However, this approach is not successful in all migraine patients, and finding a means of identifying patients who are likely to respond to amitriptyline should be a high-priority research goal.

The strengths of the current review include a comprehensive search strategy and rigorous assessment of the certainty of evidence using the latest GRADE guidance [27]. We also focus on outcomes relevant to patients, informed by an established core outcome set. We assessed the certainty of evidence using the GRADE approach [27]. While the GRADE framework presents a comprehensive framework for considering all factors that may bear on the certainty of evidence, its application is ultimately subjective, and others may come to different conclusions about the certainty of evidence. Our review does not provide any information on function, disability, or quality of life, though these outcomes are likely to correlate with monthly migraine days, responder rates and adverse events.

## Conclusions

Our systematic review and meta-analysis suggest amitriptyline to increase the proportion of patients who experience a 50% or more reduction in monthly migraine days. In fact, duration of treatment in the available studies was rather limited, whereas in real-life treatment is required for a longer period thus making tolerability more compelling. While amitriptyline may remain the first drug of choice in some patients who, for reason of comorbidities, may particularly benefit from its effect, there are no scientific data that can support to include it among the options to be mandatorily considered as first-line treatments for migraine prevention. We want to reinforce that, drugs approved and recommended for migraine prevention, must be supported by studies that adopt a high standard in terms of design and reporting.

## Supplementary Information

**Additional file 1.**

**Additional file 2.**

## Abbreviations

TCAs: Tricyclic antidepressants
FDA: Food and Drug Administration
AHS: American Headache Society
AAN: American Academy of Neurology
5-HT: 5-Hydroxytryptamine
NMDA: N-methyl-D-aspartate
CSD: Cortical spreading depression
PRISMA: Preferred Reporting Items for Systematic Reviews
COSMIG: Outcome Set for preventive intervention trials in chronic and episodic migraine
REML: Restricted maximum likelihood
AAFP: American Academy of Family Physicians
ACP: American College of Physicians
ASIM: American Society of Internal Medicine
EMA: European Medicines Agency
ECG: Electrocardiogram
CYP: Cytochrome P
NR: Not reported
NCT: Number clinical trail
MTD: Maximum tolerable dose
