# Supplementary material for: European Headache Federation (EHF) critical re-appraisal and meta-analysis of oral drugs in migraine prevention—part 1: amitriptyline
Source: J Headache Pain. 2023 Apr 11;24(1):39. doi: 10.1186/s10194-023-01573-6 (PMC10088191; doi:10.1186/s10194-023-01573-6)
Supplement: Supplementary file 1 — Additional file 1. [file 10194_2023_1573_MOESM1_ESM.pdf]

The comparative effectiveness of drugs for migraine prevention: a systematic review and network meta-analysis

Table of Contents

The comparative effectiveness of drugs for migraine prevention: a systematic review and network meta-analysis 1

Table of Contents 2

Supplement 1 – Search strategy 3

Supplement 2 – Risk of bias criteria 16

Supplement 3 – Sensitivity analysis restricted to recommended therapeutic doses of drugs 19

Supplement 4 – Table of trial characteristics 20

Supplement 5 – Comparisons and GRADE ratings for network meta-analysis of 50% or more reduction in monthly migraine days .... **Fehler! Textmarke nicht definiert.**

Supplement 6 – Pairwise meta-analyses for 50% or more reduction in monthly migraine days..... **Fehler! Textmarke nicht definiert.**

Supplement 7 – Node split plots for 50% or more reduction in monthly migraine days. **Fehler! Textmarke nicht definiert.**

Supplement 8 – Secondary analysis for 50% or more reduction in monthly migraine days (all monoclonal antibodies are grouped in one node) .... **Fehler! Textmarke nicht definiert.**

Supplement 9 – Secondary analysis for 50% or more reduction in monthly migraine days (restricted trials to those that investigated recommended therapeutic doses of drugs) .... **Fehler! Textmarke nicht definiert.**

Supplement 10 – Subgroup analyses for 50% or more reduction in monthly migraine days .... **Fehler! Textmarke nicht definiert.**

Supplement 11 – Network diagram for monthly migraine days .... **Fehler! Textmarke nicht definiert.**

Supplement 12 – Comparisons and GRADE ratings for network meta-analysis of monthly migraine days................ **Fehler! Textmarke nicht definiert.**

Supplement 13 – Pairwise meta-analyses for monthly migraine days .... **Fehler! Textmarke nicht definiert.**

Supplement 14 – Node split plots for monthly migraine days .... **Fehler! Textmarke nicht definiert.**

Supplement 15 – Network diagram for adverse events leading to discontinuation........ **Fehler! Textmarke nicht definiert.**

Supplement 16 – Comparisons and GRADE ratings for network meta-analysis of adverse events leading to discontinuation .... **Fehler! Textmarke nicht definiert.**

Supplement 17 – Pairwise meta-analyses for adverse events leading to discontinuation .... **Fehler! Textmarke nicht definiert.**

Supplement 18 – Node split plots for adverse events leading to discontinuation........... **Fehler! Textmarke nicht definiert.**

Supplement 19 – Secondary analysis for adverse events leading to discontinuation (all monoclonal antibodies are grouped in one node) .... **Fehler! Textmarke nicht definiert.**

Supplement 20 – Secondary analysis for adverse events leading to discontinuation (restricted trials to those that investigated recommended therapeutic doses of drugs) .... **Fehler! Textmarke nicht definiert.**

Supplement 1 – Search strategy
Search Strategies

PRISMA Initial Results: total number of results before duplicates removed

**Database [Platform]** Searches run August 13, 2022. *No date, language limits used.*	**Results**
OVID Medline Epub Ahead of Print, In-Process & Other Non-Indexed Citations, Ovid MEDLINE(R) Daily and Ovid MEDLINE(R) 1946 to Present [Ovid]	5,913
Embase 1974 to 2022 August 12 [Ovid]	5,983
EBM Reviews - Cochrane Central Register of Controlled Trials July 2022 [Ovid]	3,267
**TOTAL**	**15,163**

ClinicalTrials.gov Results Searched August 13, 2022.

Studies With Results | Interventional Studies | Migraine Disorders | various treatment terms [see strategies below in document]	94
**TOTAL**	**94**

ClinicalTrials.gov Search Strategies

1 Study found for: CGRP monoclonal antibodies | Studies With Results | Interventional Studies | Migraine Disorders
8 Studies found for: adrenergic beta-antagonists | Studies With Results | Interventional Studies | Migraine Disorders
1 Study found for: Calcium Channel Blocker | Studies With Results | Interventional Studies | Migraine Disorders
22 Studies found for: Anticonvulsants | Studies With Results | Interventional Studies | Migraine Disorders
39 Studies found for: Anti-Inflammatory Agents, Non-Steroidal | Studies With Results | Interventional Studies | Migraine Disorders
1 Study found for: angiotensin receptor antagonists | Studies With Results | Interventional Studies | Migraine Disorders
5 Studies found for: antidepressive agents | Studies With Results | Interventional Studies | Migraine Disorders
11 Studies found for: Botulinum Toxins, Type A | Studies With Results | Interventional Studies | Migraine Disorders
No Studies found for: coenzyme Q10 | Studies With Results | Interventional Studies | Migraine Disorders
3 Studies found for: Magnesium | Studies With Results | Interventional Studies | Migraine Disorders
3 Studies found for: Melatonin | Studies With Results | Interventional Studies | Migraine Disorders

MEDLINE(R) 1996 to August 12, 2022
Search Strategy:

#	Searches	Results
1	exp Migraine Disorders/	22110
2	(migraine or migraines or sick headache or sick headaches or hemicrania or migrainous headache or status hemicranicus).tw,kf.	28623
3	or/1-2	30802
4	("cgrp monoclonal antibodies" or "calcitonin gene-related peptide antibodies (cgrp) monoclonal antibodies (mabs)" or "cgrp mabs").tw,kf.	127
5	calcitonin gene related peptide.tw,kf.	8169

6	Calcitonin Gene-Related Peptide Receptor Antagonists/	848
7	exp Antibodies, Monoclonal/	191819
8	6 and 7	274
9	adrenergic beta-antagonists/	24650
10	("adrenergic beta antagonist*" or "adrenergic beta blocker*" or "adrenergic beta receptor blockader*" or "beta adrenergic antagonist*" or "beta adrenergic blocker*" or "beta adrenergic blocking agent*" or "beta adrenergic blocking drug" or "beta adrenergic receptor antagonist*" or "beta adrenergic receptor blockader*" or "beta adrenergic receptor blocker*" or "beta adrenoceptor antagonist*" or "beta adrenoceptor blocker*" or "beta adrenoceptor blocking agent*" or "beta adrenoceptor blocking drug*" or "beta adrenolytic" or "beta antagonist*" or "beta antiadrenergic agent*" or "beta blocker" or "beta blocking adrenergic agent*" or "beta blocking agent*" or "beta blocking drug*" or "beta receptor adrenergic blocking agent*" or "beta receptor blocker*" or "beta receptor blocking agent*" or "beta sympathicolytic agent*" or "beta sympathicolytics" or "beta sympatholytic agent*" or "betasympatholytic agent*").tw,kf.	14206
11	alprenolol/ or brimonidine tartrate, timolol maleate drug combination/ or bunolol/ or bupranolol/ or carteolol/ or carvedilol/ or dihydroalprenolol/ or iodocyanopindolol/ or labetalol/ or levobunolol/ or metipranolol/ or nadolol/ or oxprenolol/ or penbutolol/ or pindolol/ or propranolol/ or sotalol/ or timolol/	14502
12	(alprenolol or bucindolol or bunolol or bupranolol or carazolol or carpindolol or carteolol or carvedilol or cloranolol or dexpropranolol or dichlorisoprenaline or dihydroalprenolol or dilevalol or iodocyanopindolol or isamoltane or labetalol or levobunolol or metipranolol or nadolol or nifenalol or oxprenolol or penbutolol or pindolol or propranolol or sotalol or spirendolol or tertatolol or tilisolol or timolol).tw,kf.	19291
13	adrenergic beta-1 receptor antagonists/	1281
14	acebutolol/ or atenolol/ or betaxolol/ or bisoprolol/ or celiprolol/ or metoprolol/ or practolol/	6735
15	(acebutolol or atenolol or bendacalol or betaxolol or bevantolol or bisoprolol or celiprolol or cetamolol or cyanoiodopindolol or cyanopindolol or dramedilol or epanolol or esmolol or flusoxolol or landiolol or metoprolol or nebivolol or practolol or propranolol or ritodrine or salcardolol or sandoz 204545 or sotalol or talinolol or vortioxetine).tw,kf.	22643
16	Calcium Channel Blockers/	25268
17	("calcium blocker" or "calcium blocking agent*" or "calcium channel antagonist*" or "calcium channel blocker*" or "calcium channel blocking drug*" or "calcium entry blocker" or "calcium entry blocking agent*" or "calcium inhibitor" or "exogenous calcium antagonist*" or "exogenous calcium blockader*").tw,kf.	12479
18	Anticonvulsants/	40862
19	("anti convulsant agent*" or "anti convulsive agent*" or "anti convulsive drug*" or "anti epileptic*" or "anticonvulsant*" or "anticonvulsive agent*" or "anticonvulsive drug*" or "anticonvulsivum" or "antiepileptic" or "antiepileptics" or "antiepileptiform drug*").tw,kf.	34720
20	acetazolamide/ or bromides/ or cannabidiol/ or carbamazepine/ or chlormethiazole/ or clobazam/ or clonazepam/ or clorazepate dipotassium/ or diazepam/ or dimethadione/ or estazolam/ or ethosuximide/ or felbamate/ or flunarizine/ or gabapentin/ or lacosamide/ or lamotrigine/ or levetiracetam/ or lorazepam/ or magnesium sulfate/ or medazepam/ or mephenytoin/ or	57455

	mephobarbital/ or meprobamate/ or nitrazepam/ or oxcarbazepine/ or paraldehyde/ or phenobarbital/ or phenytoin/ or pregabalin/ or primidone/ or riluzole/ or thiopental/ or tiagabine/ or tiletamine/ or topiramate/ or trimethadione/ or valproic acid/ or vigabatrin/ or zonisamide/	
21	(acetazolamide or albutoin or alprazolam or ascorbate magnesium or belnacasan or brexanolone or brivaracetam or bromides or cannabidiol or cannabidivarin or carabersat or carbamazepine or carisbamate or cenobamate or chlormethiazole or clobazam or clomethiazole or clonazepam or clorazepate dipotassium or darigabat or dextromethorphan or dezinamide or diazepam or diclofenamide or dimethadione or elpetrigine or estazolam or eterobarb or ethosuximide or ethotoin or etiracetam or felbamate or fenfluramine or flunarizine or gabapentin or ganaxolone or guaifenesin or "ica 105665" or imepitoin or imidazenil or lacosamide or lamotrigine or lanicemine or levetiracetam or licarbazepine or lorazepam or loreclezole or losigamone or magnesium sulfate or medazepam or mephenytoin or mephobarbital or meprobamate or mesuximide or metharbital or midazolam or nitrazepam or oxcarbazepine or padsevonil or paraldehyde or paramethadione or pentoxyverine or perampanel or phenobarbital or phenytoin or pregabalin or primidone or progabide or remacemide or retigabine or riluzole or ropizine or rufinamide or safinamide or seletracetam or sodium bromide or stiripentol or sultiame or talampanel or thiopental or tiagabine or tiletamine or topiramate or trimethadione or valproic acid or valpromide or valrocemide or vigabatrin or vixotrigine or zaleplon or zonisamide).tw,kf.	82787
22	exp Anti-Inflammatory Agents, Non-Steroidal/	138244
23	("anti inflammatory analgesic*" or "aspirin like agent*" or "non steroid antiinflammatory agent*" or "non steroid antiinflammatory drug*" or "non steroidal anti inflammatory agent*" or "non steroidal anti inflammatory drug*" or "non steroidal antiinflammatory agent*" or "non steroidal antiinflammatory drug*" or "nonsteroid antiinflammatory agent*" or "nonsteroid antiinflammatory drug" or "nonsteroid antirheumatic agent" or "nonsteroidal anti inflammatory agent*" or "nonsteroidal anti inflammatory drug*" or "nonsteroidal antiinflammatory agent*" or "nonsteroidal antiinflammatory drug*" or "nsaid" or "nsaids").tw,kf.	36916
24	Acetaminophen/ or Antipyrine/ or Aspirin/ or Celecoxib/ or Clonixin/ or Curcumin/ or Diclofenac/ or dipyrone/ or Ibuprofen/ or Indomethacin/ or Ketoprofen/ or Ketorolac/ or Ketorolac Tromethamine/ or Mesalamine/ or Naproxen/ or salicylates/ or Sulfasalazine/ or Tolmetin/	96692
25	(acalabrutinib or aceclofenac or acemetacin or acetaminophen or acetaminosalol or acetylsalicylic acid or actarit or adalimumab or alemtuzumab or antipyrine or apremilast or ascriptin or aspirin or azathioprine or azelaic acid or balsalazide or belimumab or brimonidine or celecoxib or clonixin lysine or clonixin or curcumin or dexibuprofen or dexketoprofen or diclofenac or dipyrone or etodolac or etoricoxib or fenoprofen or flurbiprofen or ibuprofen or icosapentaenoic acid or indometacin or indomethacin or ketoprofen or ketorolac or leflunomide or lornoxicam or loxoprofen or lumiracoxib or meclofenamic acid or mefenamic acid or meloxicam or mesalamine or mesalazine or methotrexate or nabumetone or naproxen or natalizumab or nimesulide or parecoxib or phenylbutazone or piroxicam or pirprofen or rasagiline or rituximab or rofecoxib or ruxolitinib or salazosulfapyridine or salicylates or salicylic acid or satralizumab or sulfasalazine or sulindac or tenoxicam or teriflunomide or tofacitinib or tolmetin or valdecoxib).tw,kf.	203782
26	exp Angiotensin Receptor Antagonists/	24152

27	("angiotensin ii receptor antagonist*" or "angiotensin ii receptor blocker*" or "angiotensin ii receptor blocking agent*" or "angiotensin receptor antagonist" or "angiotensin receptor blocker*" or "angiotensin receptor blocking agent*").tw,kf.	12160
28	irbesartan/ or olmesartan medoxomil/ or telmisartan/ or valsartan/	6612
29	("angiotensin 1 receptor antagonist" or "angiotensin 2 receptor antagonist" or azilsartan or candesartan or eprosartan or fimasartan or irbesartan or losartan or olmesartan or sartan derivative or tasosartan or telmisartan or trv027 or valsartan).tw,kf.	17916
30	exp Antidepressive Agents/	94901
31	("anti depressant agent*" or "antidepressant*" or "antidepression drug*" or "antidepressive agent*" or "antidepressive drug*" or "neurothymoleptic agent*" or "psychoenergizer" or "thymoanaleptic" or "thymoanaleptics" or thymoleptic or thymoleptics or "thymolytic agent").tw,kf.	52365
32	Aripiprazole/ or Duloxetine Hydrochloride/ or Lithium Carbonate/ or Lithium Compounds/ or Mirtazapine/ or Moclobemide/ or Phenelzine/ or Pizotyline/ or Quetiapine Fumarate/ or Sertraline/ or Tranylcypromine/	16745
33	Bupropion/ or Citalopram/ or Fluoxetine/ or Mianserin/ or Paroxetine/ or Sulpiride/ or Trazodone/ or Tryptophan/ or Venlafaxine Hydrochloride/	38437
34	Amitriptyline/ or Clomipramine/ or Desipramine/ or Dothiepin/ or Doxepin/ or Imipramine/ or Nortriptyline/ or Opipramol/	7959
35	(agomelatine or amitriptyline or aprepitant or aripiprazole or asenapine or "botulinum toxin a" or bupropion or citalopram or clomipramine or desipramine or dothiepin or doxepin or duloxetine hydrochloride or esketamine or fluoxetine or gepirone or imipramine or indalpine or ipsapirone or lithium acetate or lithium carbonate or lithium chloride or lithium compounds or lithium salt or mianserin or mifepristone or minaprine or mirtazapine or moclobemide or monoamine oxidase inhibitor* or noradrenalin uptake inhibitor* or nortriptyline or opipramol or paroxetine or phenelzine or pizotyline or quetiapine fumarate or serotonin uptake inhibitor* or sertraline or sulpiride or tetracyclic antidepressant* or tranylcypromine or trazodone or tricyclic antidepressant* or tryptophan or venlafaxine hydrochloride).tw,kf.	84436
36	Botulinum Toxins, Type A/	10631
37	("abobotulinum toxin a" or "abobotulinumtoxin a" or "abobotulinumtoxina" or "agn 151607" or "agn151607" or "alluzience" or "ant 1207" or "ant 1401" or "ant 1403" or "ant1207" or "ant1401" or "ant1403" or "azzalure" or "bocouture" or "boe−tox" or "bont a" or "bont serotype a" or "botox" or "botulin a" or "botulin toxin a" or "botulinium a toxin" or "botulinum a exotoxin" or "botulinum a toxin" or "botulinum neurotoxin a" or "botulinum toxin a" or "btxa" or "clostridium botulinum a toxin" or "clostridium botulinum endotoxin" or "clostridium botulinum neurotoxin a" or "clostridium botulinum toxin type a" or "clostridium botulinum type a neurotoxin" or "cnt 52120" or "cnt52120" or "daxibotulinum toxin a" or "daxibotulinumtoxin a" or "daxibotulinumtoxina" or "dwp 450" or "dwp450" or "dyslor" or "dysport" or "evabotulinum toxin a" or "evabotulinumtoxin a" or "evabotulinumtoxina" or "evosyal" or "gemibotulinum toxin a" or "gemibotulinumtoxin a" or "gemibotulinumtoxina" or "gsk 1358820" or "gsk1358820" or "incobotulinum toxin a" or "incobotulinumtoxin a" or "incobotulinumtoxina" or "ipn 59011" or "ipn59011" or "jeuveau" or "letibotulinum toxin a" or "letibotulinumtoxin a" or "letibotulinumtoxina" or "meditoxin" or "mt 10109" or "mt10109" or "nabota" or "neuronox" or "nivobotulinum toxin a" or "nivobotulinumtoxin a" or "nivobotulinumtoxina" or "nt 201" or "nt201" or "nuceiva" or "oculinum" or "onabotulinum toxin a" or "onabotulinumtoxin a" or	7610

	"onabotulinumtoxina" or "onaclostox" or "pm 12759" or "pm12759" or "prabotulinum toxin a" or "prabotulinumtoxin a" or "prabotulinumtoxina" or "prosigne" or "purtox" or "qm 1114" or "qm1114" or "relabotulinum toxin a" or "relabotulinumtoxin a" or "relabotulinumtoxina" or "reloxin" or "rtt 150" or "rtt150" or "vistabel" or "vistabex" or "xeomeen" or "xeomin").tw,kf.	
38	(caomet or "coenzyme 910" or "coenzyme q 10" or "coenzyme Q10" or decorenone or mitocor or neuquinone or "quinone q 10" or ubidecarenone or ubimaior or "ubiquinone (10)" or "ubiquinone 10" or "ubiquinone 50" or ubiten).tw,kf.	4064
39	Magnesium/	24624
40	(magnesium or romag).tw,kf.	32593
41	Melatonin/	17282
42	("apl 510" or "apl510" or ceyesto or circadin or "jan 13004" or "jan13004" or "ki 1001" or "ki1001" or melatonin or melatonina or melovine or orlogin or slenyto or "sp 13004" or "sp13004" or waferest).tw,kf.	20405
43	or/4-5,8-42	696356
44	3 and 43 [migraines AND prophylaxis]	6793
45	randomized controlled trial.pt.	480050
46	controlled clinical trial.pt.	49673
47	randomized.ab.	460234
48	placebo.ab.	169835
49	drug therapy.fs.	1880993
50	randomly.ab.	294766
51	trial.ab.	482925
52	groups.ab.	1714791
53	45 or 46 or 47 or 48 or 49 or 50 or 51 or 52	3949647
54	animals/ not humans.sh.	2817192
55	53 not 54	3422601
56	44 and 55 [migraines AND prophylaxis AND RCTs]	4566
57	remove duplicates from 56	4540

OVID Medline Epub Ahead of Print, In-Process & Other Non-Indexed Citations, Ovid MEDLINE(R) Daily and Ovid MEDLINE(R) 1946 to Present

Search Strategy:

#	Searches	Results
1	exp Migraine Disorders/	30669
2	(migraine or migraines or sick headache or sick headaches or hemicrania or migrainous headache or status hemicranicus).tw,kf.	40663
3	or/1-2	44880
4	("cgrp monoclonal antibodies" or "calcitonin gene-related peptide antibodies (cgrp) monoclonal antibodies (mabs)" or "cgrp mabs").tw,kf.	167
5	calcitonin gene related peptide.tw,kf.	12349

6	Calcitonin Gene-Related Peptide Receptor Antagonists/	869
7	exp Antibodies, Monoclonal/	267646
8	6 and 7	274
9	adrenergic beta-antagonists/	41510
10	("adrenergic beta antagonist*" or "adrenergic beta blocker*" or "adrenergic beta receptor blockader*" or "beta adrenergic antagonist*" or "beta adrenergic blocker*" or "beta adrenergic blocking agent*" or "beta adrenergic blocking drug" or "beta adrenergic receptor antagonist*" or "beta adrenergic receptor blockader*" or "beta adrenergic receptor blocker*" or "beta adrenoceptor antagonist*" or "beta adrenoceptor blocker*" or "beta adrenoceptor blocking agent*" or "beta adrenoceptor blocking drug*" or "beta adrenolytic" or "beta antagonist*" or "beta antiadrenergic agent*" or "beta blocker" or "beta blocking adrenergic agent*" or "beta blocking agent*" or "beta blocking drug*" or "beta receptor adrenergic blocking agent*" or "beta receptor blocker*" or "beta receptor blocking agent*" or "beta sympathicolytic agent*" or "beta sympathicolytics" or "beta sympatholytic agent*" or "betasympatholytic agent*").tw,kf.	28363
11	alprenolol/ or brimonidine tartrate, timolol maleate drug combination/ or bunolol/ or bupranolol/ or carteolol/ or carvedilol/ or dihydroalprenolol/ or iodocyanopindolol/ or labetalol/ or levobunolol/ or metipranolol/ or nadolol/ or oxprenolol/ or penbutolol/ or pindolol/ or propranolol/ or sotalol/ or timolol/	48975
12	(alprenolol or bucindolol or bunolol or bupranolol or carazolol or carpindolol or carteolol or carvedilol or cloranolol or dexpropranolol or dichlorisoprenaline or dihydroalprenolol or dilevalol or iodocyanopindolol or isamoltane or labetalol or levobunolol or metipranolol or nadolol or nifenalol or oxprenolol or penbutolol or pindolol or propranolol or sotalol or spirendolol or tertatolol or tilisolol or timolol).tw,kf.	51527
13	adrenergic beta-1 receptor antagonists/	1328
14	acebutolol/ or atenolol/ or betaxolol/ or bisoprolol/ or celiprolol/ or metoprolol/ or practolol/	14374
15	(acebutolol or atenolol or bendacalol or betaxolol or bevantolol or bisoprolol or celiprolol or cetamolol or cyanoiodopindolol or cyanopindolol or dramedilol or epanolol or esmolol or flusoxolol or landiolol or metoprolol or nebivolol or practolol or propranolol or ritodrine or salcardolol or sandoz 204545 or sotalol or talinolol or vortioxetine).tw,kf.	54958
16	Calcium Channel Blockers/	37478
17	("calcium blocker" or "calcium blocking agent*" or "calcium channel antagonist*" or "calcium channel blocker*" or "calcium channel blocking drug*" or "calcium entry blocker" or "calcium entry blocking agent*" or "calcium inhibitor" or "exogenous calcium antagonist*" or "exogenous calcium blockader*").tw,kf.	20506
18	Anticonvulsants/	54979
19	("anti convulsant agent*" or "anti convulsive agent*" or "anti convulsive drug*" or "anti epileptic*" or "anticonvulsant*" or "anticonvulsive agent*" or "anticonvulsive drug*" or "anticonvulsivum" or "antiepileptic" or "antiepileptics" or "antiepileptiform drug*").tw,kf.	52702
20	acetazolamide/ or bromides/ or cannabidiol/ or carbamazepine/ or chlormethiazole/ or clobazam/ or clonazepam/ or clorazepate dipotassium/ or diazepam/ or dimethadione/ or estazolam/ or ethosuximide/ or felbamate/ or flunarizine/ or gabapentin/ or lacosamide/ or lamotrigine/ or levetiracetam/ or lorazepam/ or magnesium sulfate/ or medazepam/ or mephenytoin/ or	121063

	mephobarbital/ or meprobamate/ or nitrazepam/ or oxcarbazepine/ or paraldehyde/ or phenobarbital/ or phenytoin/ or pregabalin/ or primidone/ or riluzole/ or thiopental/ or tiagabine/ or tiletamine/ or topiramate/ or trimethadione/ or valproic acid/ or vigabatrin/ or zonisamide/	
21	(acetazolamide or albutoin or alprazolam or ascorbate magnesium or belnacasan or brexanolone or brivaracetam or bromides or cannabidiol or cannabidivarin or carabersat or carbamazepine or carisbamate or cenobamate or chlormethiazole or clobazam or clomethiazole or clonazepam or clorazepate dipotassium or darigabat or dextromethorphan or dezinamide or diazepam or diclofenamide or dimethadione or elpetrigine or estazolam or eterobarb or ethosuximide or ethotoin or etiracetam or felbamate or fenfluramine or flunarizine or gabapentin or ganaxolone or guaifenesin or "ica 105665" or imepitoin or imidazenil or lacosamide or lamotrigine or lanicemine or levetiracetam or licarbazepine or lorazepam or loreclezole or losigamone or magnesium sulfate or medazepam or mephenytoin or mephobarbital or meprobamate or mesuximide or metharbital or midazolam or nitrazepam or oxcarbazepine or padsevonil or paraldehyde or paramethadione or pentoxyverine or perampanel or phenobarbital or phenytoin or pregabalin or primidone or progabide or remacemide or retigabine or riluzole or ropizine or rufinamide or safinamide or seletracetam or sodium bromide or stiripentol or sultiame or talampanel or thiopental or tiagabine or tiletamine or topiramate or trimethadione or valproic acid or valpromide or valrocemide or vigabatrin or vixotrigine or zaleplon or zonisamide).tw,kf.	148034
22	exp Anti-Inflammatory Agents, Non-Steroidal/	211076
23	("anti inflammatory analgesic*" or "aspirin like agent*" or "non steroid antiinflammatory agent*" or "non steroid antiinflammatory drug*" or "non steroidal anti inflammatory agent*" or "non steroidal anti inflammatory drug*" or "non steroidal antiinflammatory agent*" or "non steroidal antiinflammatory drug*" or "nonsteroid antiinflammatory agent*" or "nonsteroid antiinflammatory drug" or "nonsteroid antirheumatic agent" or "nonsteroidal anti inflammatory agent*" or "nonsteroidal anti inflammatory drug*" or "nonsteroidal antiinflammatory agent*" or "nonsteroidal antiinflammatory drug*" or "nsaid" or "nsaids").tw,kf.	51073
24	Acetaminophen/ or Antipyrine/ or Aspirin/ or Celecoxib/ or Clonixin/ or Curcumin/ or Diclofenac/ or dipyrone/ or Ibuprofen/ or Indomethacin/ or Ketoprofen/ or Ketorolac/ or Ketorolac Tromethamine/ or Mesalamine/ or Naproxen/ or salicylates/ or Sulfasalazine/ or Tolmetin/	156025
25	(acalabrutinib or aceclofenac or acemetacin or acetaminophen or acetaminosalol or acetylsalicylic acid or actarit or adalimumab or alemtuzumab or antipyrine or apremilast or ascriptin or aspirin or azathioprine or azelaic acid or balsalazide or belimumab or brimonidine or celecoxib or clonixin lysine or clonixin or curcumin or dexibuprofen or dexketoprofen or diclofenac or dipyrone or etodolac or etoricoxib or fenoprofen or flurbiprofen or ibuprofen or icosapentaenoic acid or indometacin or indomethacin or ketoprofen or ketorolac or leflunomide or lornoxicam or loxoprofen or lumiracoxib or meclofenamic acid or mefenamic acid or meloxicam or mesalamine or mesalazine or methotrexate or nabumetone or naproxen or natalizumab or nimesulide or parecoxib or phenylbutazone or piroxicam or pirprofen or rasagiline or rituximab or rofecoxib or ruxolitinib or salazosulfapyridine or salicylates or salicylic acid or satralizumab or sulfasalazine or sulindac or tenoxicam or teriflunomide or tofacitinib or tolmetin or valdecoxib).tw,kf.	307676
26	exp Angiotensin Receptor Antagonists/	26926

27	("angiotensin ii receptor antagonist*" or "angiotensin ii receptor blocker*" or "angiotensin ii receptor blocking agent*" or "angiotensin receptor antagonist" or "angiotensin receptor blocker*" or "angiotensin receptor blocking agent*").tw,kf.	14264
28	irbesartan/ or olmesartan medoxomil/ or telmisartan/ or valsartan/	6658
29	("angiotensin 1 receptor antagonist" or "angiotensin 2 receptor antagonist" or azilsartan or candesartan or eprosartan or fimasartan or irbesartan or losartan or olmesartan or sartan derivative or tasosartan or telmisartan or trv027 or valsartan).tw,kf.	20660
30	exp Antidepressive Agents/	157396
31	("anti depressant agent*" or "antidepressant*" or "antidepression drug*" or "antidepressive agent*" or "antidepressive drug*" or "neurothymoleptic agent*" or "psychoenergizer" or "thymoanaleptic" or "thymoanaleptics" or thymoleptic or thymoleptics or "thymolytic agent").tw,kf.	73764
32	Aripiprazole/ or Duloxetine Hydrochloride/ or Lithium Carbonate/ or Lithium Compounds/ or Mirtazapine/ or Moclobemide/ or Phenelzine/ or Pizotyline/ or Quetiapine Fumarate/ or Sertraline/ or Tranylcypromine/	21662
33	Bupropion/ or Citalopram/ or Fluoxetine/ or Mianserin/ or Paroxetine/ or Sulpiride/ or Trazodone/ or Tryptophan/ or Venlafaxine Hydrochloride/	61997
34	Amitriptyline/ or Clomipramine/ or Desipramine/ or Dothiepin/ or Doxepin/ or Imipramine/ or Nortriptyline/ or Opipramol/	24431
35	(agomelatine or amitriptyline or aprepitant or aripiprazole or asenapine or "botulinum toxin a" or bupropion or citalopram or clomipramine or desipramine or dothiepin or doxepin or duloxetine hydrochloride or esketamine or fluoxetine or gepirone or imipramine or indalpine or ipsapirone or lithium acetate or lithium carbonate or lithium chloride or lithium compounds or lithium salt or mianserin or mifepristone or minaprine or mirtazapine or moclobemide or monoamine oxidase inhibitor* or noradrenalin uptake inhibitor* or nortriptyline or opipramol or paroxetine or phenelzine or pizotyline or quetiapine fumarate or serotonin uptake inhibitor* or sertraline or sulpiride or tetracyclic antidepressant* or tranylcypromine or trazodone or tricyclic antidepressant* or tryptophan or venlafaxine hydrochloride).tw,kf.	143344
36	Botulinum Toxins, Type A/	10662
37	("abobotulinum toxin a" or "abobotulinumtoxin a" or "abobotulinumtoxina" or "agn 151607" or "agn151607" or "alluzience" or "ant 1207" or "ant 1401" or "ant 1403" or "ant1207" or "ant1401" or "ant1403" or "azzalure" or "bocouture" or "boe−tox" or "bont a" or "bont serotype a" or "botox" or "botulin a" or "botulin toxin a" or "botulinium a toxin" or "botulinum a exotoxin" or "botulinum a toxin" or "botulinum neurotoxin a" or "botulinum toxin a" or "btxa" or "clostridium botulinum a toxin" or "clostridium botulinum endotoxin" or "clostridium botulinum neurotoxin a" or "clostridium botulinum toxin type a" or "clostridium botulinum type a neurotoxin" or "cnt 52120" or "cnt52120" or "daxibotulinum toxin a" or "daxibotulinumtoxin a" or "daxibotulinumtoxina" or "dwp 450" or "dwp450" or "dyslor" or "dysport" or "evabotulinum toxin a" or "evabotulinumtoxin a" or "evabotulinumtoxina" or "evosyal" or "gemibotulinum toxin a" or "gemibotulinumtoxin a" or "gemibotulinumtoxina" or "gsk 1358820" or "gsk1358820" or "incobotulinum toxin a" or "incobotulinumtoxin a" or "incobotulinumtoxina" or "ipn 59011" or "ipn59011" or "jeuveau" or "letibotulinum toxin a" or "letibotulinumtoxin a" or "letibotulinumtoxina" or "meditoxin" or "mt 10109" or "mt10109" or "nabota" or "neuronox" or "nivobotulinum toxin a" or "nivobotulinumtoxin a" or "nivobotulinumtoxina" or "nt 201" or "nt201" or "nuceiva" or "oculinum" or "onabotulinum toxin a" or "onabotulinumtoxin a" or	9189

	"onabotulinumtoxina" or "onaclostox" or "pm 12759" or "pm12759" or "prabotulinum toxin a" or "prabotulinumtoxin a" or "prabotulinumtoxina" or "prosigne" or "purtox" or "qm 1114" or "qm1114" or "relabotulinum toxin a" or "relabotulinumtoxin a" or "relabotulinumtoxina" or "reloxin" or "rtt 150" or "rtt150" or "vistabel" or "vistabex" or "xeomeen" or "xeomin").tw,kf.	
38	(caomet or "coenzyme 910" or "coenzyme q 10" or "coenzyme Q10" or decorenone or mitocor or neuquinone or "quinone q 10" or ubidecarenone or ubimaior or "ubiquinone (10)" or "ubiquinone 10" or "ubiquinone 50" or ubiten).tw,kf.	5376
39	Magnesium/	69283
40	(magnesium or romag).tw,kf.	66468
41	Melatonin/	22186
42	("apl 510" or "apl510" or ceyesto or circadin or "jan 13004" or "jan13004" or "ki 1001" or "ki1001" or melatonin or melatonina or melovine or orlogin or slenyto or "sp 13004" or "sp13004" or waferest).tw,kf.	28086
43	or/4-5,8-42	1183314
44	3 and 43 [migraines AND prophylaxis]	9301
45	randomized controlled trial.pt.	574929
46	controlled clinical trial.pt.	94985
47	randomized.ab.	571967
48	placebo.ab.	230792
49	drug therapy.fs.	2519979
50	randomly.ab.	389035
51	trial.ab.	612187
52	groups.ab.	2393254
53	45 or 46 or 47 or 48 or 49 or 50 or 51 or 52	5433260
54	animals/ not humans.sh.	5002581
55	53 not 54	4733125
56	44 and 55 [migraines AND prophylaxis AND RCTs]	5941
57	remove duplicates from 56	5913

EBM Reviews - Cochrane Central Register of Controlled Trials July 2022

Search Strategy:

#	Searches	Results
1	exp Migraine Disorders/	3020
2	(migraine or migraines or sick headache or sick headaches or hemicrania or migrainous headache or status hemicranicus).tw,kw.	9024
3	1 or 2	9137
4	("cgrp monoclonal antibodies" or "calcitonin gene-related peptide antibodies (cgrp) monoclonal antibodies (mabs)" or "cgrp mabs").tw,kw.	13
5	calcitonin gene related peptide.tw,kw.	1062
6	Calcitonin Gene-Related Peptide Receptor Antagonists/	81

7	exp Antibodies, Monoclonal/	15809
8	6 and 7	42
9	adrenergic beta-antagonists/	4417
10	("adrenergic beta antagonist*" or "adrenergic beta blocker*" or "adrenergic beta receptor blockader*" or "beta adrenergic antagonist*" or "beta adrenergic blocker*" or "beta adrenergic blocking agent*" or "beta adrenergic blocking drug" or "beta adrenergic receptor antagonist*" or "beta adrenergic receptor blockader*" or "beta adrenergic receptor blocker*" or "beta adrenoceptor antagonist*" or "beta adrenoceptor blocker*" or "beta adrenoceptor blocking agent*" or "beta adrenoceptor blocking drug*" or "beta adrenolytic" or "beta antagonist*" or "beta antiadrenergic agent*" or "beta blocker" or "beta blocking adrenergic agent*" or "beta blocking agent*" or "beta blocking drug*" or "beta receptor adrenergic blocking agent*" or "beta receptor blocker*" or "beta receptor blocking agent*" or "beta sympathicolytic agent*" or "beta sympathicolytics" or "beta sympatholytic agent*" or "betasympatholytic agent*").tw,kw.	6202
11	alprenolol/ or "brimonidine tartrate, timolol maleate drug combination"/ or bunolol/ or bupranolol/ or carteolol/ or carvedilol/ or dihydroalprenolol/ or iodocyanopindolol/ or labetalol/ or levobunolol/ or metipranolol/ or nadolol/ or oxprenolol/ or penbutolol/ or pindolol/ or propranolol/ or sotalol/ or timolol/	6240
12	(alprenolol or bucindolol or bunolol or bupranolol or carazolol or carpindolol or carteolol or carvedilol or cloranolol or dexpropranolol or dichlorisoprenaline or dihydroalprenolol or dilevalol or iodocyanopindolol or isamoltane or labetalol or levobunolol or metipranolol or nadolol or nifenalol or oxprenolol or penbutolol or pindolol or propranolol or sotalol or spirendolol or tertatolol or tilisolol or timolol).tw,kw.	10888
13	adrenergic beta-1 receptor antagonists/	215
14	acebutolol/ or atenolol/ or betaxolol/ or bisoprolol/ or celiprolol/ or metoprolol/ or practolol/	4201
15	(acebutolol or atenolol or betaxolol or bevantolol or bisoprolol or celiprolol or cetamolol or cyanoiodopindolol or cyanopindolol or esmolol or landiolol or metoprolol or nebivolol or practolol or propranolol or ritodrine or sotalol or talinolol or vortioxetine).tw,kw.	13586
16	Calcium Channel Blockers/	2876
17	("calcium blocker" or "calcium blocking agent*" or "calcium channel antagonist*" or "calcium channel blocker*" or "calcium channel blocking drug*" or "calcium entry blocker" or "calcium entry blocking agent*" or "calcium inhibitor" or "exogenous calcium antagonist*" or "exogenous calcium blockader*").tw,kw.	3325
18	Anticonvulsants/	2440
19	("anti convulsant agent*" or "anti convulsive agent*" or "anti convulsive drug*" or "anti epileptic*" or "anticonvulsant*" or "anticonvulsive agent*" or "anticonvulsive drug*" or "anticonvulsivum" or "antiepileptic" or "antiepileptics" or "antiepileptiform drug*").tw,kw.	4495
20	acetazolamide/ or bromides/ or cannabidiol/ or carbamazepine/ or chlormethiazole/ or clobazam/ or clonazepam/ or clorazepate dipotassium/ or diazepam/ or dimethadione/ or estazolam/ or ethosuximide/ or felbamate/ or flunarizine/ or gabapentin/ or lacosamide/ or lamotrigine/ or levetiracetam/ or lorazepam/ or magnesium sulfate/ or medazepam/ or mephenytoin/ or mephobarbital/ or meprobamate/ or nitrazepam/ or oxcarbazepine/ or paraldehyde/ or phenobarbital/	11619

	or phenytoin/ or pregabalin/ or primidone/ or riluzole/ or thiopental/ or tiagabine/ or tiletamine/ or topiramate/ or trimethadione/ or valproic acid/ or vigabatrin/ or zonisamide/	
21	(acetazolamide or albutoin or alprazolam or ascorbate magnesium or belnacasan or brexanolone or brivaracetam or bromides or cannabidiol or cannabidivarin or carabersat or carbamazepine or carisbamate or cenobamate or chlormethiazole or clobazam or clomethiazole or clonazepam or clorazepate dipotassium or darigabat or dextromethorphan or dezinamide or diazepam or diclofenamide or dimethadione or elpetrigine or estazolam or eterobarb or ethosuximide or ethotoin or etiracetam or felbamate or fenfluramine or flunarizine or gabapentin or ganaxolone or guaifenesin or "ica 105665" or imepitoin or imidazenil or lacosamide or lamotrigine or lanicemine or levetiracetam or licarbazepine or lorazepam or loreclezole or losigamone or magnesium sulfate or medazepam or mephenytoin or mephobarbital or meprobamate or mesuximide or metharbital or midazolam or nitrazepam or oxcarbazepine or padsevonil or paraldehyde or paramethadione or pentoxyverine or perampanel or phenobarbital or phenytoin or pregabalin or primidone or progabide or remacemide or retigabine or riluzole or ropizine or rufinamide or safinamide or seletracetam or sodium bromide or stiripentol or sultiame or talampanel or thiopental or tiagabine or tiletamine or topiramate or trimethadione or valproic acid or valpromide or valrocemide or vigabatrin or vixotrigine or zaleplon or zonisamide).tw,kw.	34246
22	exp Anti-Inflammatory Agents, Non-Steroidal/	21883
23	("anti inflammatory analgesic*" or "aspirin like agent*" or "non steroid antiinflammatory agent*" or "non steroid antiinflammatory drug*" or "non steroidal anti inflammatory agent*" or "non steroidal anti inflammatory drug*" or "non steroidal antiinflammatory agent*" or "non steroidal antiinflammatory drug*" or "nonsteroid antiinflammatory agent*" or "nonsteroid antiinflammatory drug" or "nonsteroid antirheumatic agent" or "nonsteroidal anti inflammatory agent*" or "nonsteroidal anti inflammatory drug*" or "nonsteroidal antiinflammatory agent*" or "nonsteroidal antiinflammatory drug*" or "nsaid" or "nsaids").tw,kw.	11038
24	Acetaminophen/ or Antipyrine/ or Aspirin/ or Celecoxib/ or Clonixin/ or Curcumin/ or Diclofenac/ or dipyrone/ or Ibuprofen/ or Indomethacin/ or Ketoprofen/ or Ketorolac/ or Ketorolac Tromethamine/ or Mesalamine/ or Naproxen/ or salicylates/ or Sulfasalazine/ or Tolmetin/	19158
25	(acalabrutinib or aceclofenac or acemetacin or acetaminophen or acetaminosalol or acetylsalicylic acid or actarit or adalimumab or alemtuzumab or antipyrine or apremilast or ascriptin or aspirin or azathioprine or azelaic acid or balsalazide or belimumab or brimonidine or celecoxib or clonixin lysine or clonixin or curcumin or dexibuprofen or dexketoprofen or diclofenac or dipyrone or etodolac or etoricoxib or fenoprofen or flurbiprofen or ibuprofen or icosapentaenoic acid or indometacin or indomethacin or ketoprofen or ketorolac or leflunomide or lornoxicam or loxoprofen or lumiracoxib or meclofenamic acid or mefenamic acid or meloxicam or mesalamine or mesalazine or methotrexate or nabumetone or naproxen or natalizumab or nimesulide or parecoxib or phenylbutazone or piroxicam or pirprofen or rasagiline or rituximab or rofecoxib or ruxolitinib or salazosulfapyridine or salicylates or salicylic acid or satralizumab or sulfasalazine or sulindac or tenoxicam or teriflunomide or tofacitinib or tolmetin or valdecoxib).tw,kw.	70589
26	exp angiotensin receptor antagonists/	4178
27	("angiotensin ii receptor antagonist*" or "angiotensin ii receptor blocker*" or "angiotensin ii receptor blocking agent*" or "angiotensin receptor antagonist" or "angiotensin receptor blocker*" or "angiotensin receptor blocking agent*").tw,kw.	3582
28	irbesartan/ or olmesartan medoxomil/ or telmisartan/ or valsartan/	2080

29	("angiotensin 1 receptor antagonist" or "angiotensin 2 receptor antagonist" or azilsartan or candesartan or eprosartan or fimasartan or irbesartan or losartan or olmesartan or sartan derivative or tasosartan or telmisartan or trv027 or valsartan).tw,kw.	8620
30	exp antidepressive agents/	15881
31	("anti depressant agent*" or "antidepressant*" or "antidepression drug*" or "antidepressive agent*" or "antidepressive drug*" or "neurothymoleptic agent*" or "psychoenergizer" or "thymoanaleptic" or "thymoanaleptics" or thymoleptic or thymoleptics or "thymolytic agent").tw,kw.	13906
32	Aripiprazole/ or Duloxetine Hydrochloride/ or Lithium Carbonate/ or Lithium Compounds/ or Mirtazapine/ or Moclobemide/ or Phenelzine/ or Pizotyline/ or Quetiapine Fumarate/ or Sertraline/ or Tranylcypromine/	4101
33	Bupropion/ or Citalopram/ or Fluoxetine/ or Mianserin/ or Paroxetine/ or Sulpiride/ or Trazodone/ or Tryptophan/ or Venlafaxine Hydrochloride/	6608
34	Amitriptyline/ or Clomipramine/ or Desipramine/ or Dothiepin/ or Doxepin/ or Imipramine/ or Nortriptyline/ or Opipramol/	3410
35	(agomelatine or amitriptyline or aprepitant or aripiprazole or asenapine or "botulinum toxin a" or bupropion or citalopram or clomipramine or desipramine or dothiepin or doxepin or duloxetine hydrochloride or esketamine or fluoxetine or gepirone or imipramine or indalpine or ipsapirone or lithium acetate or lithium carbonate or lithium chloride or lithium compounds or lithium salt or mianserin or mifepristone or minaprine or mirtazapine or moclobemide or monoamine oxidase inhibitor* or noradrenalin uptake inhibitor* or nortriptyline or opipramol or paroxetine or phenelzine or pizotyline or quetiapine fumarate or serotonin uptake inhibitor* or sertraline or sulpiride or tetracyclic antidepressant* or tranylcypromine or trazodone or tricyclic antidepressant* or tryptophan or venlafaxine hydrochloride).tw,kw.	25811
36	Botulinum Toxins, Type A/	1835
37	("abobotulinum toxin a" or "abobotulinumtoxin a" or "abobotulinumtoxina" or "agn 151607" or "agn151607" or "alluzience" or "ant 1207" or "ant 1401" or "ant 1403" or "ant1207" or "ant1401" or "ant1403" or "azzalure" or "bocouture" or "boe−tox" or "bont a" or "bont serotype a" or "botox" or "botulin a" or "botulin toxin a" or "botulinium a toxin" or "botulinum a exotoxin" or "botulinum a toxin" or "botulinum neurotoxin a" or "botulinum toxin a" or "btxa" or "clostridium botulinum a toxin" or "clostridium botulinum endotoxin" or "clostridium botulinum neurotoxin a" or "clostridium botulinum toxin type a" or "clostridium botulinum type a neurotoxin" or "cnt 52120" or "cnt52120" or "daxibotulinum toxin a" or "daxibotulinumtoxin a" or "daxibotulinumtoxina" or "dwp 450" or "dwp450" or "dyslor" or "dysport" or "evabotulinum toxin a" or "evabotulinumtoxin a" or "evabotulinumtoxina" or "evosyal" or "gemibotulinum toxin a" or "gemibotulinumtoxin a" or "gemibotulinumtoxina" or "gsk 1358820" or "gsk1358820" or "incobotulinum toxin a" or "incobotulinumtoxin a" or "incobotulinumtoxina" or "ipn 59011" or "ipn59011" or "jeuveau" or "letibotulinum toxin a" or "letibotulinumtoxin a" or "letibotulinumtoxina" or "meditoxin" or "mt 10109" or "mt10109" or "nabota" or "neuronox" or "nivobotulinum toxin a" or "nivobotulinumtoxin a" or "nivobotulinumtoxina" or "nt 201" or "nt201" or "nuceiva" or "oculinum" or "onabotulinum toxin a" or "onabotulinumtoxin a" or "onabotulinumtoxina" or "onaclostox" or "pm 12759" or "pm12759" or "prabotulinum toxin a" or "prabotulinumtoxin a" or "prabotulinumtoxina" or "prosigne" or "purtox" or "qm 1114" or "qm1114" or "relabotulinum toxin a" or "relabotulinumtoxin a" or "relabotulinumtoxina" or "reloxin" or "rtt 150" or "rtt150" or "vistabel" or "vistabex" or "xeomeen" or "xeomin").tw,kw.	3672

38	(caomet or "coenzyme 910" or "coenzyme q 10" or "coenzyme Q10" or decorenone or mitocor or neuquinone or "quinone q 10" or ubidecarenone or ubimaior or "ubiquinone (10)" or "ubiquinone 10" or "ubiquinone 50" or ubiten).tw,kw.	1080
39	Magnesium/	1229
40	(magnesium or romag).tw,kw.	7739
41	Melatonin/	1328
42	("apl 510" or "apl510" or ceyesto or circadin or "jan 13004" or "jan13004" or "ki 1001" or "ki1001" or melatonin or melatonina or melovine or orlogin or slenyto or "sp 13004" or "sp13004" or waferest).tw,kw.	3246
43	or/4-5,8-42	197886
44	3 and 43	3267

Supplement 2 – Risk of bias criteria

**Bias from the randomization process**	
Issues to consider:<br>Random sequence generation<br>Allocation concealment	
**Definitely low risk of bias**	Trials that assign participants to alternative interventions using a randomly generated sequence and maintain allocation concealment.<br><br>Examples of methods for developing a randomly generated allocation sequence include a random number generator, random number table, coin tossing, shuffling cards or envelopes, and throwing dice. If a trial is described as 'randomized' without any additional details related to how the allocation sequence was developed, we will assume that the allocation sequence was appropriately developed.<br><br>Examples of methods for maintaining allocation concealment include using central allocation via a computer or phone system, pharmacy-controlled allocation, opaque sealed envelopes, and sequentially numbered drug containers.<br><br>*Note that an explicit description of random sequence generation is not necessary for a rating of low risk of bias.*
**Probably low risk of bias**	Trials in which healthcare providers were blind to the intervention but which provide no information on allocation concealment.<br><br>*Note that an explicit description of random sequence generation is not necessary for a rating of probably low risk of bias.*
**Probably high risk of bias**	Trials in which healthcare providers were not blind to the intervention and which provide no information on allocation concealment.<br><br>Trials in which there are substantial baseline differences between trial arms that suggest a problem with the randomization process but there are no other limitations related to randomization.
**Definitely high risk of bias**	Trials in which allocation is by judgment of the clinician, by preference of the participant, by availability of the intervention, based on the results of a laboratory test, or other non-random rules (e.g., birthdate, etc.).<br><br>Trials in which investigators enrolling participants could possibly foresee the arm to which each subsequent patient would be randomized, such as allocation using an open allocation schedule (e.g. a list of random numbers), assignment envelopes used without appropriate safeguards (e.g. use of unsealed, non-opaque or not sequentially numbered envelopes), alternation between arms, case record number, or any other explicitly unconcealed procedure, rate as high risk.
**Bias due to deviations from the intended intervention**	
Issues to consider:<br>Blinding of healthcare providers/clinicians and participants<br>Imbalances in cointerventions or behaviors	

**Definitely low risk of bias**	Trials in which healthcare providers are blind to the intervention administered and in which there are no significant differences in administered co-interventions.<br><br>Trials that are described as double or triple blind.
**Probably low risk of bias**	
**Probably high risk of bias**	Trials in which healthcare providers are not blind to the intervention administered.<br><br>Trials in which healthcare providers are blind to the intervention administered but there are significant differences in administered co-interventions that suggests that blinding may have been compromised.<br><br>Trials in which healthcare providers are described as being blind to the intervention but allocation concealment was inadequate.
**Definitely high risk of bias**	Trials in which healthcare providers are not blind to the intervention and in which there are significant differences in administered co-interventions.
**Bias due to missing data**	
Issues to consider:<br>Missing outcome measures<br>Loss to follow-up	
**Definitely low risk of bias**	Trials in which missing outcome data (including outcome data that has been imputed) < 10%.
**Probably low risk of bias**	Trials in which missing outcome data (including outcome data that has been imputed) is between 10% to 15% and missing outcome data is unlikely to be related to the true outcome and there is no imbalance in numbers of or reasons for missing data across intervention groups.
**Probably high risk of bias**	Trials in which missing outcome data (including outcome data that has been imputed) is between 10% to 15% and missing outcome data is likely to be related to the true outcome or there are imbalances in numbers of or reasons for missing data across intervention groups.
**Definitely high risk of bias**	Trials in which missing outcome data (including outcome data that has been imputed) > 15%.
**Bias due to measurement of the outcome**	
Issues to consider:<br>Blinding of outcome adjudicators<br>Objectivity of outcome<br><br>*Note that the judgments may differ across outcomes.*	
**Definitely low risk of bias**	Trials in which patients are blind to the intervention and in which outcomes are patient-reported.<br><br>Trials in which outcomes are measured by a third-party (investigator or clinician) and in which the third-party is blind to the intervention.<br><br>Trials in which the outcomes are objective (e.g., mortality, hospitalization).<br><br>Trials that are described as double or triple blind.
**Probably low risk of bias**	

**Probably high risk of bias**	
**Definitely high risk of bias**	Trials in which patients are not blind and in which outcomes are patient-reported (e.g., ACQ).<br><br>Trials in which outcome adjudicators are not blind and the outcomes are not objective (e.g., adverse events leading to discontinuation).
**Bias in selection of the reported results**	
Issues to consider:<br>Selective reporting of timepoints<br>Selective reporting of outcome measures<br><br>*Note that we are only interested in selective reporting for the outcomes for which we are extracting data.*<br><br>*Note that the judgments may differ across outcomes.*	
**Definitely low risk of bias**	Results for outcomes that were analyzed and reported according to a pre-specified statistical analysis plan or protocol (including the timepoint for the measurement of the outcome).
**Probably low risk of bias**	Results for outcomes that were analyzed and reported but that were not prespecified in a statistical analysis plan or protocol but the timepoint at which results are reported is consistent with the timepoint for other outcomes in the trial report or there is little reason to believe the outcome was selectively reported.<br><br>Please note that outcomes that were not prespecified in a protocol or statistical analysis plan and that are reported in the trial preprint or publication should be rated at probably low risk of bias unless there are other important reasons to suspect that results for those outcomes were selectively reported (e.g., results are presented at timepoints that don't match the timepoints reported for other outcomes).
**Probably high risk of bias**	Results for outcomes that were analyzed and reported but that were not prespecified in a statistical analysis plan or protocol but the timepoint at which results are reported is not consistent with the timepoint for other outcomes in the trial report or there are other reasons to believe that the outcome is selectively reported.
**Definitely high risk of bias**	Results for outcomes that were analyzed and reported for which there are inconsistencies with the statistical analysis plan or protocol. These inconsistencies may include outcome measures of interest or the timepoints for the measurement of outcomes.

Supplement 3 – Sensitivity analysis restricted to recommended therapeutic doses of drugs

We performed a sensitivity analysis in which we restricted trial arms to only those that tested recommended therapeutic doses of drugs.

Drugs	Minimum dose
Amitriptyline	10 mg/day
Beta-blocker	Bisoprolol: 5 mg/day<br>Propranolol: 40 mg/day
Calcium channel blocker	Flunarizine: 5 mg/day<br>Cinnarizine: 15 mg/day
Carisbamate	100 mg/day
Eptinezumab	100 mg/month
Erenumab	70 mg/month
Fremanezumab	225 mg/month or 675 mg/3 months
Gabapentin	100 mg/day
Galcanezumab	120 mg/month
Gepant	Atogepant: 10 mg/day<br>Rimegepant: 75 mg/day<br>Telcagepant: 280 mg/day
Oxcarbezapine	300 mg/day
Topiramate	50 mg/day
Valproate	500 mg/day

Ducros A, de Gaalon S, Roos C, Donnet A, Giraud P, Guégan-Massardier E, Lantéri-Minet M, Lucas C, Mawet J, Moisset X, Valade D, Demarquay G. Revised guidelines of the French headache society for the diagnosis and management of migraine in adults. Part 2: Pharmacological treatment. Rev Neurol (Paris). 2021 Sep;177(7):734-752. doi: 10.1016/j.neurol.2021.07.006. Epub 2021 Jul 30. PMID: 34340810.

Supplement 4 – Table of trial characteristics

Study	Trial Name	Registration	Funding	Country	Age	% Male	Duration of Migraine (years)	% Aura	% With Previous Prophylaxis	Migraine/ Headache Days Per Month	Interventions
**Ailani, 2021 (1)**	ADVANCE	NCT03777059	Allergan	United States	41.6	11.2	NR	NR	70.3	7.4	Atogepant (10mg): 10 mg/day for 12 weeks, oral<br>Atogepant (30mg): 30 mg/day for 12 weeks, oral<br>Atogepant (60mg): 60 mg/day for 12 weeks, oral<br>Placebo
**Ashina, 2020 (2)**	PROMISE-1	NCT02559895	H. Lundbeck A/S	United States	39.8	15.7	17.4	NR	NR	8.6	Eptinezumab (30mg): 30 mg, q12w, IV<br>Eptinezumab (100mg): 100 mg, q12w, IV<br>Eptinezumab (300mg): 300 mg, q12w, IV<br>Placebo
**Ashina, 2022 (3)**	DELIVER	NCT04418765	H. Lundbeck A/S	Belgium, Bulgaria, Czechia, Denmark, Finland, France, Georgia, Germany, Hungary, Italy, Poland, Russia, Slovakia, Spain, Sweden, United Kingdom, United States	43.8	10.1	14.6	29.6	100.0	13.8	Eptinezumab (100mg): 100 mg, day 0 and week 12, IV<br>Eptinezumab (300mg): 300 mg, day 0 and week 12, IV<br>Placebo
**Bigal, 2015 (4)**	NR	NCT02025556	Teva Pharmaceuticals	United States	41.2	12.1	19.0	NR	29.0	11.4	Fremanezumab (225mg):225 mg/day q28d for 12 weeks, SC<br>Fremanezumab (675mg): 675mg q28d for 12 weeks, SC<br>Placebo
**Bigal, 2016 (5)**	NR	NCT02021773	Teva Pharmaceuticals	United States	40.7	14.1	18.3	NR	40.3	16.8	Fremanezumab (675/225mg): 675 mg loading dose, 225 mg/day q28d for 12 weeks, SC<br>Fremanezumab (900mg): 900 mg, q28d for 12 weeks, SC<br>Placebo
**Bostani, 2013 (6)**	NR	IRCT201102055729N2	Kermanshah University of Medical Sciences	Iran	32.1	31.7	6.4	17.3	NR	6.8	Valproate: 200 mg/day for 12 weeks, oral<br>Cinnarizine: 25 mg/day for 12 weeks, oral

**Brandes, 2004 (7)**	MIGR-002	NCT00231595	Johnson and Johnson Pharmaceutical Research and Development	Canada, United States	38.9	13.2	NR	NR	NR	6.5	Topiramate (50mg): 50 mg titrated over 8 weeks, maintained for 18 weeks, oral<br>Topiramate (100mg): 100 mg titrated over 8 weeks, maintained for 18 weeks, oral<br>Topiramate (200mg): 200 mg titrated over 8 weeks, maintained for 18 weeks, oral<br>Placebo
**Cady, 2009 (8)**	NR	NCT00109083	Johnson and Johnson Pharmaceutical Research and Development	Germany, Spain, United States	41.3	14.6	19.2	46.8	NR	8.1	Carisbamate (100mg): 100 mg/day titrated over 2 weeks, maintained for 12 weeks, oral<br>Carisbamate (300mg): 300 mg/day titrated over 2 weeks, maintained for 12 weeks, oral<br>Carisbamate (600mg): 600 mg/day titrated over 2 weeks, maintained for 12 weeks, oral<br>Placebo
**Camporeale, 2018 (9)**	NR	NCT02614287	Eli Lilly and Company	Belgium, Canada, France, Hungary, United States	42.0	17.4	20.8	NR	62.6	10.6	Galcanezumab (120mg): 120 mg/month for 52 weeks, SC<br>Galcanezumab (240 mg): 240 mg/month for 52 weeks, SC
**Chowdhury, 2021 (10)**	TOP-PRO	CTRI/2019/05/018997	None	India	33.0	4.6	6.0	4.6	NR	17.4	Topiramate: 100 mg/day titrated over 4 weeks, maintained for 20 weeks, oral<br>Propranolol: 80 mg/day titrated over 4 weeks, maintained for 20 weeks, oral
**Couch, 1979 (11)**	NR	NR	Merck Laboratories	United States	NR	16.0	NR	NR	NR	NR	Amitriptyline: 100 mg/day or MTD, titrated over 4 weeks, maintained for 4 weeks, oral<br>Placebo
**Couch, 2011 (12)**	NR	NR	Merck, Sharp, and Dohme Research Laboratories	United States	34.9	19.0	NR	NR	NR	NR	Amitriptyline: 100 mg/day or MTD, titrated over 4 weeks, maintained for 12 weeks, oral<br>Placebo
**Croop, 2021 (13)**	NR	NCT03732638	Biohaven Pharmaceuticals	United States	41.2	17.0	NR	40.0	NR	10.1	Rimegepant: 75 mg every other day for 12 weeks, oral<br>Placebo
**Detke, 2018 (14)**	REGAIN	NCT02614261	Eli Lilly and Company	Argentina, Canada, Czech Republic, Germany, Israel, Italy, Mexico, Netherlands, Spain, Taiwan, United Kingdom, United States	41.0	15.0	21.1	54.3	77.8	19.5	Galcanezumab (120mg): 120 mg, q4w for 12 weeks, SC<br>Galcanezumab (240mg): 240 mg, q4w for 12 weeks, SC<br>Placebo

**Diener, 1996 (15)**	NR	NR	NR	NR	39.0	22.0	19.0	26.2	NR	4.0	Propranolol: 120 mg/day for 12 weeks, oral<br>Placebo
**Diener, 2002 (16)**	NR	NR	Janssen	Belgium, Denmark, France, Germany, Italy, Portugal, Spain, Switzerland	37.0 *	18.6	10.0*	28.2	NR	NR	Flunarizine (5mg): 5 mg/day for 16 weeks, oral<br>Flunarizine (10mg): 10 mg/day for 16 weeks (placebo administered on 2/7 days), oral<br>Propranolol: 160 mg/day titrated over 1 weeks and maintained for 8 weeks, oral
**Diener, 2004 (17)**	MIGR-003	NCT00236561	Johnson & Johnson Pharmaceutical Research and Development	Australia, Denmark, Finland, France, Germany, Italy, Korea, Netherlands, South African, Spain, Sweden, Taiwan, United Kingdom	40.9	20.3	NR	NR	NR	6.1	Topiramate (100mg): 100 mg/day titrated over 8 weeks and maintained for 18 weeks, oral<br>Topiramate (200mg): 200 mg/day titrated over 8 weeks and maintained for 18 weeks, oral<br>Propranolol (160mg): 160 mg/day titrated over 8 weeks and maintained for 18 weeks, oral<br>Placebo
**Diener, 2007 (18)**	PROMPT	NR	Janssen-Cilag EMEA	Austria, Belgium, Bulgaria, Czech Republic, Denmark, France, Germany, Greece, Hungary, Ireland, Italy, Norway, Poland, Portugal, Russia, Saudi Arabia, Slovenia, Spain, Switzerland, Turkey, UK	39.8	13.0	NR	NR	NR	8.9	Topiramate:100mg/day or MTD, titrated over 4 weeks and maintained for 12 weeks, oral<br>Placebo
**Dodick, 2007(19)**	NR	NR	Ortho-McNeil Janssen	United States	38.2	14.7	9.2	NR	NR	17.1	Topiramate: 100 mg/day or MTD, titrated over 4 weeks maintained for 12 weeks, oral<br>Placebo
**Dodick, 2009 (20)**	NR	NR	Ortho-McNeil Janssen	United States	38.8	15.1	NR	NR	NR	7.3	Topiramate: 100 mg/day or MTD, titrated over 4 weeks and maintained for 22 weeks, oral<br>Amitriptyline: 100 mg/day or MTD titrated over 4 weeks and maintained for 22 weeks, oral
**Dodick, 2014 (21)**	NR	NCT01625988	Arteaus Therapeutics	United States	41.4	15.2	NR	41.5	NR	6.9	Galcanezumab: 150 mg, q2w for 12 weeks, SC<br>Placebo
**Dodick, 2014 (22)**	NR	NCT01772524	Alder Biopharmaceuticals	United States	38.8	18.4	NR	NR	NR	8.6	Eptinezumab: 1000 mg once for 12 weeks, IV<br>Placebo

**Dodick, 2018 (23)**	NR	NCT02629861	Teva Pharmaceuticals	Canada, Czech Republic, Finland, Israel, Japan, Poland, Russia, Spain, United States	41.8	15.2	20.2	NR	20.8	9.1	Fremanezumab (225mg): 225 mg, q4w for 12 weeks, SC<br>Fremanezumab (675mg): 675 mg once for 12 weeks, SC<br>Placebo
**Dodick, 2018 (24)**	ARISE	NCT02483585	Amgen	Denmark, France, Greece, Portugal, Russia, Spain, Switzerland, United States	42.0	14.7	21.0	50.3	46.1	8.3	Erenumab: 70 mg, q4w for 12 weeks, SC<br>Placebo
**Dodick, 2019 (25)**	NR	NCT02275117	Alder Biopharmaceuticals	Australia, Georgia, New Zealand, United States	36.6	13.2	17.9	NR	45.6	16.5	Eptinezumab (10mg): 10 mg, once for 12 weeks, IV<br>Eptinezumab (30mg): 30 mg, once for 12 weeks, IV<br>Epitinezumab (100mg): 100 mg, once for 12 weeks, IV<br>Eptinezumab (300mg): 300 mg, once for 12 weeks, IV<br>Placebo
**Ferrari, 2019 (26)**	FOCUS	NCT03308968	Teva Pharmaceuticals	Belgium, Czechia, Denmark, Finland, France, Germany, Italy, Netherlands, Poland, Spain, Sweden, Switzerland, United Kingdom, United States	46.2	16.5	24.9	NR	100.0	14.2	Fremanezumab (monthly): 225 mg q4w, SC<br>Fremanezumab (quarterly): 675 mg once, SC<br>Placebo
**Freitag, 2002 (27)**	NR	NR	Abbott Laboratories	United States	40.5	21.1	20.2	36.3	20.6	4.3	Valproate: 500-1000 mg/day, for 12 weeks, oral<br>Placebo
**Ghasami, 2009 (28)**	NR	NR	Arak University of Medical Sciences	Iran	NR	31.8	NR	NR	NR	NR	Valproate (15 mg/kg): 1-3 pills/day for 24 weeks, oral<br>Propranolol/antidepressant: 40-80mg/day propranolol with 50mg amitriptyline or 25-50mg/day nortriptyline
**Ghobadi, 2013 (29)**	NR	NR	NR	NR	47.0	16.7	NR	NR	NR	15.0	Propranolol: 40 mg/day for 24 weeks, oral<br>Nimodipine: 30 mg/day for 24 weeks, oral
**Goadsby, 2017 (30)**	STRIVE	NR	Novartis, Amgen	United States	40.5	21.1	20.2	36.3	20.6	4.3	Erenumab (70mg): 70 mg, q4w for 24 weeks, SC<br>Erenumab (140mg): 140 mg, q4w for 24 weeks, SC<br>Placebo
**Goadsby, 2020 (31)**	NR	NCT02638103	Teva Pharmaceuticals	Canada, Czechia, Finland, Israel, Japan, Poland, Russian Confederation, Spain, United States	43.5	13.0	21.6	NR	23.9	13.4	Fremanezumab (q12w): 675mg q12w for 52 weeks, SC<br>Fremanezumab (675/225 q4w chronic): 675 mg loading dose, 225 q4w for 52 weeks, SC<br>Fremanezumab (225mg q4w episodic): 225 mg q4w for 52 weeks, SC
**Goadsby, 2020 (32)**	NR	NCT02848326	Allergan	United States	40.1	13.5	19.4	22.3	28.1	7.7	Atogepant (10mg): 10 mg/day for 12 weeks, oral<br>Atogepant (30mg): 30 mg/day for 12 weeks, oral<br>Atogepant (60mg): 60 mg/day for 12 weeks, oral<br>Atogepant (30mg B.I.D): 30mg twice/day for 12 weeks, oral<br>Atogepant (60mg B.I.D): 60 mg twice/day for 12

												weeks, oral<br>Placebo
**Goncalves, 2016(33)**	NR	NCT01357031	Fundação de Amparo a Pesquisa de São Paulo	Brazil		36.9	24.6	22.2	16.1	NR	7.3	Amitriptyline: 25 mg/day for 12 weeks<br>Placebo
**Hesami, 2017(34)**	NR	IRCT2012070310178N1	NR	Iran		36.0	10.2	NR	NR	NR	10.3	Pregabalin (100mg): 100mg/day for 12 weeks, oral<br>Valproate (400mg): 400 mg/day for 12 weeks, oral
**Ho, 2014(35)**	NR	NCT00797667	Merck & Co.	United States		41.3	16.0	NR	46.6	29.1	8.2	Telcagepant (280 mg): 280mg/day for 12 weeks, oral<br>Telcagepant (560 mg): 560mg/day for 12 weeks, oral<br>Placebo
**Holroyd, 2010 (36)**	NR	NCT00910689	National Institutes of Health	United States		38.2	21.0	15.0	22.0	NR	8.5	Propranolol: 180 mg titrated over 12 weeks or MTD, offered nadolol if propranolol was not tolerated, maintained for 12 weeks, oral<br>Placebo
**Hu, 2022 (37)**	PERSIST	NCT03963232	Eli Lilly and Company	China, India, Russia		37.0	26.2	12.6	NR	44.6	6.3	Galcanezumab: 240 mg loading dose, 120 mg q4w for 12 weeks, SC<br>Placebo
**Kalita, 2013 (38)**	NR	NR	NR	India		31.9	19.7	7.3	6.3	NR	10.8	Valproate: 500-1000mg/day or MTD for 22 weeks, oral<br>Amitriptyline: 25-50mg/day or MTD for 22 weeks, oral
**Klapper, 1997 (39)**	NR	NR	Abbott Laboratories	United States		40.8	11.0	21.6	40.3	53.0	NR	Valproate (500mg): 500 mg/day, titrated over 4 weeks, maintained for 8 weeks, oral<br>Valproate (1000mg): 1000 mg/day, titrated over 4 weeks, maintained for 8 weeks, oral<br>Valproate (1500mg): 1500 mg/day, titrated over 4 weeks, maintained for 8 weeks, oral<br>Placebo
**Lipton, 2011 (40)**	INTREPID	NCT00212810	Ortho-Mcneil Janssen	NR		40.3	10.9	NR	NR	NR	11.7	Topiramate: 100 mg/day titrated over 6 weeks, maintained for 20 weeks, oral<br>Placebo

**Lipton, 2020 (41)**	PROMISE-2	NCT02974153	H. Lundbeck A/S	Belgium, Czechia, Denmark, Georgia, Germany, Hungary, Italy, Russia, Slovakia, Ukraine, United Kingdom, United States	40.5	11.8	18.1	NR	NR	16.1	Eptinezumab (100mg): 100 mg, day 0 and week 12, IV<br>Eptinezumab (300mg): 300 mg, day 0 and week 12, IV<br>Placebo
**Lucking, 1988 (42)**	Trial 1	NR	NR	NR	42.1	19.5	NR	NR	NR	NR	Flunarizine: 10 mg/day for 16 weeks, oral<br>Propranolol: 120mg/day titrated over 2 weeks, maintained for 14 weeks, oral
**Lucking, 1988 (42)**	Trial 2	NR	NR	NR	42.1	19.5	NR	NR	NR	NR	Flunarizine: 10 mg/day for 16 weeks, oral<br>Propranolol: 120mg/day titrated over 2 weeks, maintained for 14 weeks, oral
**Luo, 2012 (43)**	NR	NR	National Natural Science Foundation of China	China	43.0	28.6	4.5	NR	NR	4.5	Flunarizine (5mg): 5 mg/day for 52 weeks, oral<br>Topiramate: 100mg/day or MTD for 52 weeks<br>Flunarizine and Topiramate: 5 mg/day of flunarizine with 25-100 mg/day of topiramate for 52 weeks
**Mansoureh, 2008 (44)**	NR	NR	NR	NR	34.1	19.2	NR	NR	NR	7.2	Cinnarizine: 75 mg/day for 12 weeks, oral<br>Valproate: 600 mg/day for 12 weeks, oral
**Mathew, 1981 (45)**	NR	NR	NR	NR	34.0	9.5	NR	NR	NR	NR	Propranolol: 160mg/day or MTD for 28 weeks, oral<br>Amitriptyline: 50-75mg or MTD for 28 weeks, oral
**Mathew, 1995 (46)**	NR	NR	Abbott Laboratories	United States	45.6	22.4	25.0	27.0	NR	6.1	Valproate: 750 mg/day titrated over 4 weeks, maintained for 8 weeks, oral<br>Placebo
**Mathew, 2001 (47)**	NR	NR	NR	NR	39.6	17.2	20.8	43.7	NR	4.9	Gabapentin: 2400 mg/day titrated over 4 weeks, maintained for 8, oral<br>Placebo
**Mei 2004 (48)**	NR	NR	NR	Italy	39.2	45.8	NR	19.4	NR	5.5	Topiramate: 100mg/day titrated over 4 weeks maintained for 12, oral<br>Placebo
**Misra, 2013 (49)**	NR	NR	NR	NR	32.0*	22.8	5.0*	4.0	NR	6.1	Valproate: 500-750 mg/day for 24 weeks, oral<br>Amitriptyline: 25-50 mg/day for 24 weeks, oral
**Mulleners, 2020 (50)**	CONQUER	NR	Eli Lilly and Company	Belgium, Canada, Czechia, France, Germany, Hungary, Japan, Korea Republic, Netherlands, Spain, United Kingdom, United States	45.8	14.1	23.3	44.2	100.0	13.2	Galcanezumab: 240 mg loading dose, 120 mg q4w for 12 weeks, SC<br>Placebo
**Reuter, 2018 (51)**	LIBERTY	NCT03096834	Novartis	Australia, Austria, Belgium, Czech Republic, Denmark, Finland, France, Germany, Greece, Italy, Norway,	44.4	18.7	NR	35.4	100.0	9.3	Erenumab: 70 mg q4w for 12 weeks, SC<br>Placebo

				Netherlands, Spain, Sweden, Switzerland, United Kingdom							
**Reuter, 2022 (52)**	HER-MES	NCT03828539	Novartis	Germany	40.7	14.2	21.9	34.27	40.7	10.4	Erenumab: 140mg q4w for 24 weeks, SC<br>Topiramate: 50–100 mg/day for 24 weeks, oral
**Sakai, 2019 (53)**	NR	NCT02630459	Amgen, Novartis	Japan	NR	15.8	NR	26.1	66.3	7.8	Erenumab (28mg): 28 mg, q4w for 24 weeks, SC<br>Erenumab (70mg): 70 mg, q4w for 24 weeks, SC<br>Erenumab (140mg): 140 mg, q4w for 24 weeks, SC<br>Placebo
**Sakai, 2020 (54)**	NR	NCT02959177	Eli Lilly and Company	Japan	44.1	15.7	21.4	NR	60.6	8.7	Galcanezumab (120mg): 120 mg, q4w for 24 weeks, SC<br>Galcanezumab (240mg): 240 mg, q4w for 24 weeks, SC<br>Placebo
**Sakai, 2021 (55)**	NR	NCT03303079	Otsuka Pharmaceutical	Japan, Korea	42.8	13.7	18.7	NR	21.0	15.7	Fremanezumab (monthly) 675mg loading dose, 225mg q4w for 12 weeks, SC<br>Fremanezumab (quarterly): 675mg once for 12 weeks, SC<br>Placebo
**Sakai, 2021 (56)**	NR	NCT03303092	Otsuka Pharmaceutical	Japan, Korea	43.5	15.4	19.9	NR	19.3	8.8	Fremanezumab (monthly): 225 mg q4w, SC<br>Fremanezumab (quarterly): 3 doses of 225 mg at baseline, SC<br>Placebo
**Sargent, 1985 (57)**	NR	NR	NR	NR	30.0	21.0	20.0	NR	NR	NR	Propranolol: 120mg/day for 12 weeks<br>Valproate: 1100mg/day for 12 weeks<br>Placebo

**Silberstein, 2004 (58)**	NR	NR	Johnson & Johnson Pharmaceutical Research and Development	NR	40.4	11.3	NR	NR	NR	6.5	Topiramate (50mg): 50 mg/day titrated over 8 weeks, maintained for 18 weeks, oral<br>Topiramate (100mg): 100 mg/day, titrated over 8 weeks, maintained for 18 weeks, oral<br>Topiramate (200mg): 200 mg/day titrated over 8 weeks, maintained for 18 weeks, oral<br>Placebo
**Silberstein, 2006 (59)**	NR	NR	Ortho-Mcneil Neurologics	United States	40.5	14.2	NR	35.6	NR	4.9	Topiramate: 200 mg/day, titrated over 8 weeks, maintained for 12 weeks, oral<br>Placebo
**Silberstein, 2008 (60)**	NR	NR	Novartis	United States	40.5	15.3	NR	NR	NR	NR	Oxcarbazepine: 1200 mg/day titrated over 6 weeks, maintained for 8 weeks, oral<br>Placebo
**Silberstein, 2009 (61)**	NR	NCT00210912	Ortho-McNeil Janssen	United States	38.2	NR	NR	NR	NR	NR	Topiramate: 100 mg/day titrated over 4 weeks, maintained for 12 weeks, oral<br>Placebo
**Silberstein, 2012 (62)**	NR	NCT00772031	National Institute of Neurological Disorders and Stroke	United States	42.0 *	10.0	NR	NR	NR	18.0	Propranolol: total dose of 240 mg/day for 6 months, oral<br>Placebo
**Silberstein, 2013 (63)**	NR	NCT00742209	GlaxoSmithKline	Canada, United States	39.2	18.0	NR	NR	NR	9.2	Gabapentin (1200mg): 1200 mg/day for 20 weeks, oral<br>Gabapentin (1800mg): 1800 mg/day for 20 weeks, oral<br>Gabapentin (2400mg): 2400 mg/day for 20 weeks, oral<br>Gabapentin (3000mg): 3000 mg/day for 20 weeks, oral<br>Placebo
**Silberstein, 2017 (64)**	NR	NCT02621931	Teva Pharmaceuticals	Canada, Czechia, Finland, Israel, Poland, Russia, Spain, United States,	40.7	12.3	19.9	NR	21.2	16.2	Fremanezumab (quarterly): 675 mg q8w for 12 weeks, SC<br>Fremanezumab (monthly): 675 mg loading dose, 225 q4w for 12 weeks, SC<br>Placebo
**Skljarevski, 2018 (65)**	EVOLVE-2	NCT02614196	Eli Lilly and Company	Argentina, Czechia, Germany, Israel, Korea, Mexico, Netherlands, Spain, Taiwan, United Kingdom, United States	41.9	14.6	20.6	NR	66.2	9.1	Galcanezumab (120mg): 120 mg q4w 24 weeks, SC<br>Galcanezumab (240mg): 240 mg, q4w for 24 weeks, SC<br>Placebo

**Skljarevski, 2018 (66)**	NR	NCT02163993	Eli Lilly and Company	United States	40.2	17.1	NR	NR	NR	6.7	Galcanezumab (5mg): 5 mg, q4w for 12 weeks, SC<br>Galcenezumab (50mg): 50 mg, q4w for 12 weeks SC<br>Galcanezumab (120mg): 120 mg, q4w for 12 weeks, SC<br>Galcanezumab (300mg): 300 mg, q4w for 12 weeks, SC<br>Placebo
**Sorensen, 1991 (67)**	NR	NR	Janssen Pharmaceuticals	Denmark	42.0	20.8	17.0*	26.2	54.4	NR	Flunarizine: 10 mg/day for 16 weeks, oral<br>Metoprolol: 200 mg/day for 16 weeks, oral
**Sudilovsky, 1987(68)**	NR	NR	NR	United States	39.3	24.5	20.7	NR	NR	5.3	Nadalol (80mg): 80 mg/day for 12 weeks, oral<br>Nadolol (160mg): 160 mg/day for 12 weeks, oral<br>Propranolol: 160 mg/day for 12 weeks, oral
**Sun, 2016 (69)**	NR	NCT01952574	Amgen	Canada, United States, Denmark, Finland, Germany, Norway, Sweden, Portugal	41.1	19.5	20.4	NR	57.6	8.7	Erenumab (7mg): 7 mg, q4w for 12 weeks, SC<br>Erenumab (21mg): 21 mg, q4w for 12 weeks, SC<br>Erenumab (70mg): 70 mg, q4w for 12 weeks, SC<br>Placebo
**Takeshima, 2021 (70)**	NR	NCT03812224	Amgen	Japan	44.4	13.0	NR	NR	77.4	12.1	Erenumab: 70 mg q4w for 24 weeks, SC<br>Placebo
**Tepper, 2017 (71)**	NR	NCT02066415	Amgen	Canada, Czech Republic, Denmark, Finland, Germany, Norway, Poland, Sweden, UK, United States	42.1	17.2	21.7	41.4	NR	18	Erenumab (70mg): 70mg q4w for 12 weeks, SC<br>Erenumab (140mg): 140mg q4w for 12 weeks, SC<br>Placebo
**van de Ven, 1997 (72)**	NR	NR	Merck KGaA	Belgium, France, The Netherlands, Spain	38.7	17.7	NR	22.6	NR	5.5	Bisoprolol (5mg): 5 mg/day for 12 weeks, oral<br>Bisoprolol (10mg): 10 mg/day for 12 weeks, oral<br>Placebo
**Wang, 2021 (73)**	EMPOwER	NCT03333109	Novartis	NR	37.5	18.1	11.7	69.9	53.2	9.3	Erenumab (70mg): 70 mg, q4w for 12 weeks, SC<br>Erenumab (140mg): 140 mg, q4w for 12 weeks, SC<br>Placebo
