# Supplementary material for: European Headache Federation (EHF) critical re-appraisal and meta-analysis of oral drugs in migraine prevention—part 1: amitriptyline
Source: J Headache Pain. 2023 Apr 11;24(1):39. doi: 10.1186/s10194-023-01573-6 (PMC10088191; doi:10.1186/s10194-023-01573-6)

Supplement 1: Sensitivity analysis using the Paule-Mandel heterogeneity estimator

50% or more reduction in monthly migraine days

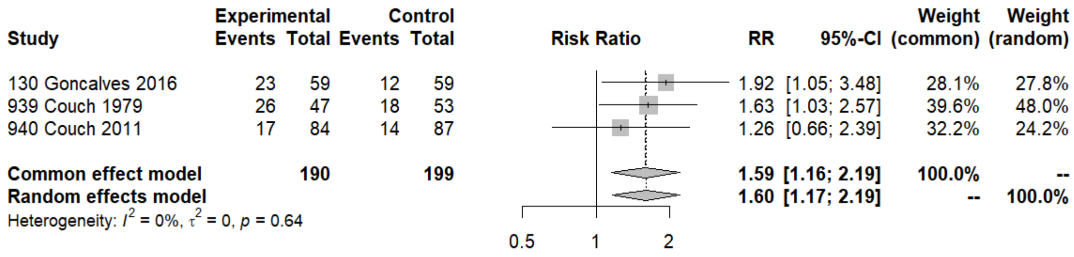

Adverse events leading to discontinuation

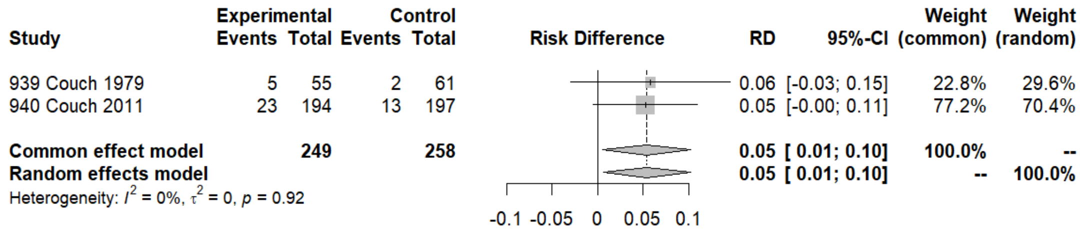

Supplement: Supplementary file 2 — Additional file 2. [file 10194_2023_1573_MOESM2_ESM.pdf]
